# Roles of the Immune/Methylation/Autophagy Landscape on Single-Cell Genotypes and Stroke Risk in Breast Cancer Microenvironment

**DOI:** 10.1155/2021/5633514

**Published:** 2021-08-19

**Authors:** Jun-yi Wu, Jun Qin, Lei Li, Kun-dong Zhang, Yi-sheng Chen, Yang Li, Tao Jin, Jun-ming Xu

**Affiliations:** Department of General Surgery, Shanghai General Hospital, Shanghai Jiaotong University School of Medicine, 200080, China

## Abstract

This study sought to perform integrative analysis of the immune/methylation/autophagy landscape on breast cancer prognosis and single-cell genotypes. Breast Cancer Recurrence Risk Score (BCRRS) and Breast Cancer Prognostic Risk Score (BCPRS) were determined based on 6 prognostic IMAAGs obtained from the TCGA-BRCA cohort. BCRRS and BCPRS, respectively, were used to construct a risk prediction model of overall survival and progression-free survival. Predictive capacity of the model was evaluated using clinical data. Analysis showed that BCRRS is associated with a high risk of stroke. In addition, PPI and drug-ceRNA networks based on differences in BCPRS were constructed. Single cells were genotyped through integrated scRNA-seq of the TNBC samples based on clustering results of BCPRS-related genes. The findings of this study show the potential regulatory effects of IMAAGs on breast cancer tumor microenvironment. High AUCs of 0.856 and 0.842 were obtained for the OS and PFS prognostic models, respectively. scRNA-seq analysis showed high expression levels of adipocytes and adipose tissue macrophages (ATMs) in high BCPRS clusters. Moreover, analysis of ligand-receptor interactions and potential regulatory mechanisms were performed. The LINC00276&MALAT1/miR-206/FZD4-Wnt7b pathway was also identified which may be useful in future research on targets against breast cancer metastasis and recurrence. Neural network-based deep learning models using BCPRS-related genes showed that these genes can be used to map the tumor microenvironment. In summary, analysis of IMAAGs, BCPRS, and BCRRS provides information on the breast cancer microenvironment at both the macro- and microlevels and provides a basis for development of personalized treatment therapy.

## 1. Introduction

Breast cancer (BRCA) is the most common cancer in women worldwide, accounting for about 25% of all female malignancies [1]. Despite advances in diagnosis and treatment, a high number of cases are diagnosed at distant metastatic sites presenting a challenge in treatment of the cancer [2, 3]. Therefore, molecular biomarkers for guiding individualized treatment and for improving the overall prognosis of breast cancer in patients are urgently needed. These biomarkers may be useful in the development of highly effective treatment options in breast cancer [4].

In the current era of precision medicine, high-throughput technology provides an opportunity to develop tumor prognostic biomarkers from different sources. These markers include Immune, Methylated, and Autophagy-Associated Genes (IMAAGs) which are potential prognostic markers in breast cancer [5–8]. Autophagy is essential in maintaining integrity of the cytoplasm and genome. In addition, it is implicated in the occurrence and development of tumors at several levels [ 3, 9]. During cancer progression, autophagy actively degrades proteins and organelles increasing the nutrient reservoir of the tumor, thus promoting tumor proliferation and invasion [10, 11]. Moreover, previous studies report that autophagy-related genes can be used as prognostic markers for breast cancer [5].

On the other hand, m6A-RNA methylation is an important internal modification in eukaryotic cells. Studies report that expression and gene changes in the m6A regulatory factors are associated with malignant tumor progression and abnormal immune regulation [12–14]. Moreover, modifications in the pattern of individual tumor m6A can predict cancer stage, subtype, genetic variation, and patient prognosis. Furthermore, m6A methylation-related genes are potential molecular markers of breast cancer prognosis [6, 7]. In addition, immune cells are shown to be involved in tumor progression [15–18]. Previous studies report that the immune characteristics of breast cancer are related with clinical features. The expression profile of immune-related genes may affect specific subtypes of breast cancer [19–21]. Evaluation of tumor immunophenotypes is an important complementary indicator of the TNM (Primary Tumor, Regional Lymph Nodes, and Distant Metastasis) stage, recurrence, and mortality [22–27].

Recent studies report that IMAAGs play a synergistic role in the tumor microenvironment [28, 29]. It was reported that m6A modification may affect the stability of autophagy-related gene transcripts and m6A methylation-related proteins can lead to tumor immune escape and development [29–32]. This implies that highly coordinated interaction exists between IMAAGs. However, no specific markers based on IMAAGs have been comprehensively applied to explore the breast cancer microenvironment and aid in prognosis. Therefore, a detailed analysis of the effect of IMAAGs on tumors will provide further knowledge on TME antitumor immune responses and guide on the development of more effective treatment options [6, 33]. Several studies report that IMAAGs are implicated in the malignant progression of breast cancer [34]. However, no study has conducted a comprehensive analysis of IMAAGs to explore their clinical significance.

Malignant differentiation of BRCA cells in the tumor microenvironment is affected by several factors [35, 36]. Single-cell transcriptomic analysis offers the opportunity to characterize cellular states and their transitions by simultaneously exploring the integrated nature of the genomes of entire tumor samples at microscopic resolution [37]. Ordering such comprehensive tumor-constituting cells into trajectories helps in understanding tumor cell subsets and the related tumorigenic and malignant transgression pathways [38]. Recent advances in single-cell analysis methods provide a more comprehensive way to explore molecular changes at the cellular level [39]. Moreover, cell-type-specific ligand-receptor complexes can be predicted by a database of the curated complexes (http://www.cellphonedb.org/) [40]. Those methods could be used to find a series of reliable prognostic markers and reveal new targets for the treatment of illness.

Therefore, a molecular and cellular map at microlevels was constructed in the current study by integrating these predictions with spatial in situ analysis. The relationship between IMAAGs and the breast cancer microenvironment has also been systematically analyzed.

## 2. Materials and Methods

### 2.1. Data Retrieval and Processing

Data sources are presented in Supplementary Table 1. Transcriptome, Copy Number Variation (CNV), and Single Nucleotide Polymorphism (SNP) data and clinical data related to breast cancer (BRCA) were downloaded from The Cancer Genome Atlas (TCGA) database. Transcriptome data were normalized using R software using library-size normalization. Autophagy-related genes were retrieved from the Human Autophagy Database (http://www.autophagy.lu/) according to previous studies [41]. Moreover, 16 m6A RNA methylation regulators with available expression data were obtained from the TCGA datasets. After that, immune-related genes were acquired from the shared data in IMMPORT (https://www.immport.org/shared/genelists). Besides, the mRNAsi index used for matching to the TCGA breast cancer dataset was obtained from a previous study [42].

The scRNA-seq data (accession number GSE118389) of a total of 1534 cells in six fresh TNBC tumors were obtained from the Gene Expression Omnibus (GEO, http://www.ncbi.nlm.nih.gov/geo/) database [43]. Samples with unavailable clinical information were excluded. The final dataset included 934 BRCAs from the TCGA cohort and 194 BRCAs from the clinical cohort.

### 2.2. Study Participants

Clinical data were obtained from 194 breast cancer patients attending the Shanghai General Hospital. According to clinical follow-up and medical history records, survival data and disease characteristics were obtained. All participants provided informed consent to participate in the study. This study was conducted in compliance with the principles of the Declaration of Helsinki. The study was approved by the Institutional Ethics Review Board of the Shanghai General Hospital (no. 2020KY211). Radiotherapy was prescribed for all patients undergoing conservative surgery, and it was recommended based on the risk of local recurrence after mastectomy following adjuvant chemotherapy.

### 2.3. Identification of Autophagy, Methylation, and Immune Associated Genes

The random forest algorithm was used to screen out genes related to breast cancer prognosis from IMAAGs. In addition, the randomForestSRC algorithm was used to rank the importance of prognostic-related genes. The random forest model was then used to screen 210 genes potentially related to the prognosis of breast cancer. A total of 19 genes that were highly correlated with prognosis were identified through single-factor Cox regression. All confounding factors were considered during the study. We have taken into account all confounding factors as much as possible. We hope to screen the most critical IMAAGs through this study. Thereafter, LASSO and multivariate Cox methods were adopted to reduce the dimensions and identify independent prognostic factors. IMAAGs that were correlated with highest risk of death were then selected to establish a multivariate Cox prognosis model [44]. Training and control group sample size was in a 7 : 3 ratio. BRCA overall survival (OS) and progression-free survival (PFS) nomogram prediction models were constructed based on the TCGA dataset and clinical data. Nomograms were validated using receiver operating characteristic (ROC) curves [45, 46]. The C-index was used to evaluate the performance of the models [47]. Decision curve analysis was used to evaluate clinical application of the nomograms [48]. Net income was calculated following a previously published method [49].

### 2.4. Tumor Purity Analysis and Immune Cellular Fraction Estimates

As per the previous research, the TCGA-BRCA RNA sequence TPM data was used as the input then the single-sample Gene Set Enrichment Analysis (ssGSEA) employed to score the enrichment of immune cell type meta genes, as described in the GSVA package of the R software [50–52]. Afterwards, *z*-scoring was performed on the data based on the prediction of infiltration and enrichment of the immune cells. The *z*-score of the data was determined based on prediction of infiltration and enrichment of immune cells. Unsupervised cluster analysis was used to identify different modification patterns in immune cells and for classification of samples for further investigation. The number of clusters and their stability were determined by the consensus clustering algorithm [53]. The ConsensusClusterPlus package was used to perform the cluster analysis with 1000 replicates to ensure stability of the classification [54]. Tumor purity score was estimated using the ESTIMATE method as described previously [55, 56].

### 2.5. Enrichment Analysis

The Gene Set Variation Analysis for Microarray and RNA-seq data (GSVA) and Gene Set Enrichment Analysis (GSEA) were performed to explore the Gene Ontology (GO) of biological processes and KEGG pathways associated with the Breast Cancer Prognostic Risk Score (BCPRS) [52, 57]. The “c2.cp.kegg.v6.2.-symbols” and “c5.all.v6.2.symbols” gene sets were retrieved from the MSigDB database for GSVA analysis. GSEA was used to explore the potential mechanisms associated with BCPRS using JAVA.

### 2.6. Quantitative Real-Time PCR (qPCR)

Total RNA was extracted from cell cultures using the Mini-BEST Universal RNA Extraction kit (TaKaRa, Kyoto, Japan). cDNA synthesis was then performed using Prime-Script RT Master Mix (TaKaRa). qPCR assays were performed using SYBR Green Master Mix (TaKaRa) in the PCR LightCycler480 system (Roche Diagnostics, Basel, Switzerland).

### 2.7. Construction of WGCNA

Transcriptome data from TCGA-BRCA was analyzed using the Weighted Gene Coexpression Network Analysis (WGCNA) method. Setting the power supply at 7 ensures a higher scale independence (close to 0.9), and lower average connectivity (close to 0) could be guaranteed. A hierarchical clustering dendrogram of a Topological Overlap Measure (TOM) matrix was constructed using the average distance with a minimum threshold of 30 and a merged cutting height of 0.25. Expression units of similar genes were then grouped into different gene modules. Cytoscope3.8 was used to visualize the coexpression network. The “igraph” package was used to determine the degrees of the module. DAVID (http://david-d.ncifcrf.gov) and GOplot tools were used for the KEGG pathway enrichment and GO function enrichment analyses of the genes screened by the WGCNA method [58].

### 2.8. Identification of DEGs between BCPRS Phenotypes

To explore BCPRS-related genes, patients were divided into two groups with different BCPRS phenotypes based on the BCPRS score. The Bayesian method in the limma R package was then used to determine Differentially Expressed Genes (DEGs) between the two groups (*p* < 0.05).

### 2.9. Construction of Drug-ceRNA Network

The miRcode database was used to explore interactions between DE-lncRNAs and DE-miRNAs as previously reported [59, 60]. Correlation between differentially expressed mRNAs (DEMs) and DE-miRNAs was explored using the miRWalk3.0 database and the miRTarBase (Version 7.0), which contains validated miRNA target interactions from various experiments [61]. The LncMAP tool was used to determine Spearman correlation coefficients between lncRNA expression levels and the IC50 values of 24 drugs. A possible drug-lncRNA network was then constructed based on the prediction of the LncMAP database.

### 2.10. TNBC scRNA-seq Data Analysis

A total of 1535 cells in six fresh TNBC tumors were included in this analysis. Patients with triple-negative breast cancer have a poor prognosis and are associated with a high risk of recurrence and metastasis; therefore, studying this dataset facilitates exploration of the potential role of BCPRS-related genes. The Seurat package in R 3.6.3 was used for quality control [62]. Gene expression levels of the remaining 1266 cells were normalized using the Seurat package. PCA was performed to identify significantly available dimensions with a *p* value < 0.05 [63]. The Uniform Manifold Approximation and Projection (UMAP) algorithm was applied for dimensionality reduction with 20 initial PCs and for performing cluster classification analysis across all cells [64]. Different cell clusters were identified and annotated using the singleR package based on the composition patterns of the marker genes and were then corrected using the CellMarker tool [65, 66]. The Monocle 2 algorithm was used to construct single-cell pseudotime trajectories of the TNBC scRNA-seq data [67]. In addition, clustering analysis was performed based on six BCPRS genes (HEY1, INFA13, NKX2-3, NR2F1, POU5F1, and YY1). DEGs between clusters 2 and 3 of adipocytes were defined as marker genes. Cell-to-cell interaction analysis was performed using the CellPhoneDB database [40]. Significant cell-to-cell interactions were determined using *p* value < 0.01.

### 2.11. Neural Network-Based Deep Learning Framework and Statistical Analysis

Neural networks were constructed using python (version 3.6) software to predict breast cancer cell types [68]. All cells were randomly assigned to a training set and a testing set with a 7 : 3 ratio. The parameter settings are the same as in the previous article [37, 68]. All statistical analyses were performed using the GraphPad Prism (version 7.0) software and R (version 3.5.3) software. The Kaplan-Meier method was used to calculate the overall survival rate, as described previously [69]. Conditional Survival (CS) was defined as the probability that the patient would survive for “*y*” years because they had survived for “*x*” years [69–73].

## 3. Results

### 3.1. Identification of Different Immunity, Methylation, and Autophagy-Related Genes

**Figure 1:**
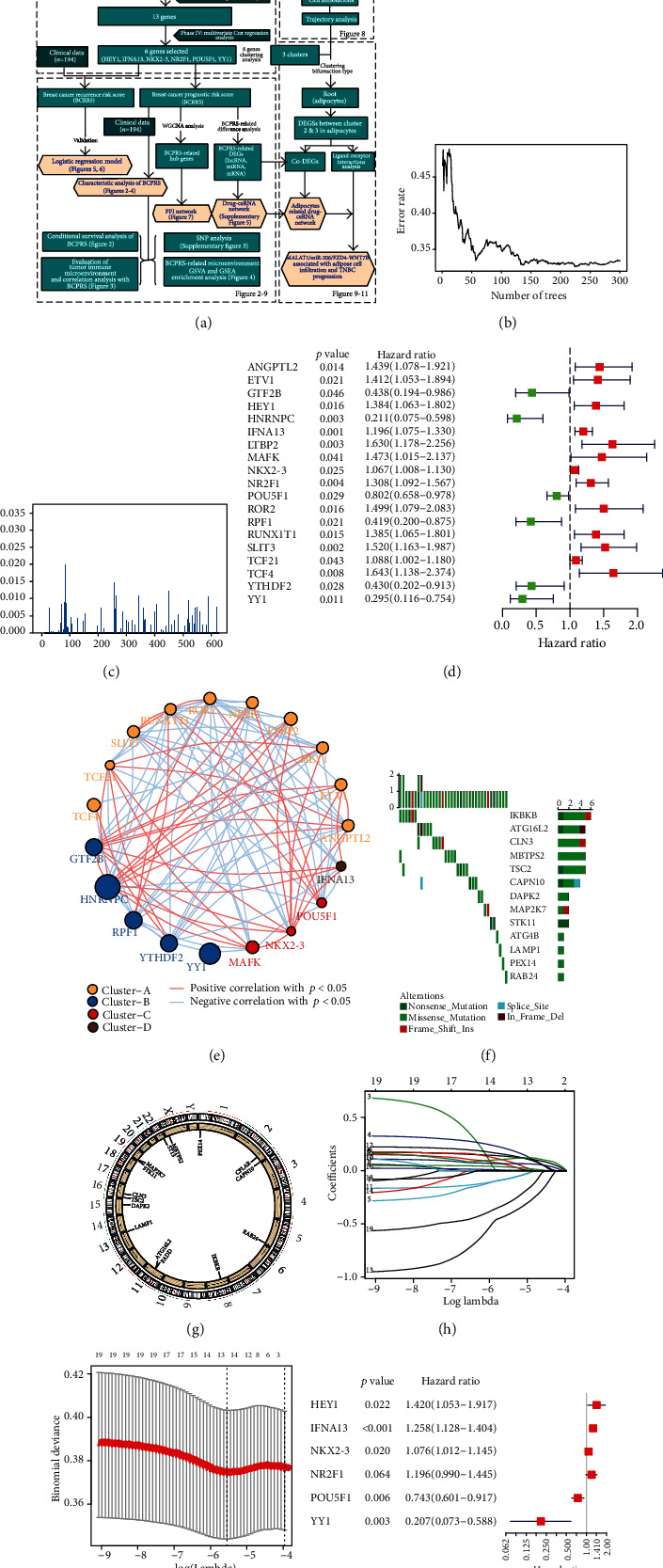
Identification and screening of different immune, methylation, and autophagy-related genes (IMAAGs). (a) The experimental design. RNA-seq and clinical data from 1109 BRCA samples were retrieved from TCGA database. A total of immune-related genes, 16 m6A methylation-related genes, and 222 autophagy-related genes were included in this study. Breast Cancer Recurrence Risk Score (BCRRS) and Breast Cancer Prognostic Risk Score (BCPRS) systems were constructed based on these genes. The BRCA nomogram prediction model, potential drug-ceRNA network, PPI network, and single-cell analysis were then constructed. (b) Data error rate of the classification tree function. (c) Important values of the genes in the random forest model. (d) A forest chart showing factors selected from the single-factor COX regression analysis (*p* < 0.05). (e) Interaction of the immune, methylation, and autophagy-related genes. The size of each cell represents the survival effect of each gene. The correlation coefficient estimated by Spearman's correlation analysis is represented by the thickness of each line. Red represents a positive correlation whereas blue indicates a negative correlation. (f) Waterfall plot of tumor somatic mutations showed that genes related to the breast (IKBKB, ATG16L2, CLN3, MBTPS2, TSC2, and CAPN10) had a high frequency of mutations. (g) Breast cancer OS-associated genes (CLN3, TSC2, DAPK2, LAMP1, ATG16L2, FADD, IKBKB, RAB24, CAPN10, CFLAR, PEX14, MBTPS2, ST13, MAP2K7, and STK11) in the positions of CNV on 23 chromosomes based on TCGA-BRCA dataset. (h, i) Key genes selected using the LASSO regression model using the minimum criterion of 5-fold cross-validation. Generation of coefficient outline based on the log (lambda) sequence, where the optimal lambda acquires the characteristics of the 6 nonzero coefficients. (j) A forest chart showing the factors selected from the multivariate COX regression analysis (*p* < 0.05).

The study design is presented in Figure 1(a) and Supplementary Figure 1. Firstly, RNA-seq and clinical data from 1109 BRCA samples were downloaded from the TCGA database. After that, 386 immune-related genes, 16 m6A methylation-related genes, and 222 autophagy-related genes were obtained. Random forest analysis was used to identify 210 genes related to the prognosis of breast cancer (Figures 1(b) and 1(c)). Moreover, 19 genes associated with the prognosis of breast cancer were identified using single-factor COX regression (Figure 1(d)).

The gene regulatory network described the interaction between immune-related, methylation-related, and autophagy-related genes as well as their impact on the prognosis of patients with breast cancer (Figure 1(e)). The results showed that some of the genes related to the prognosis of breast cancer (IKBKB, ATG16L2, CLN3, MBTPS2, TSC2, and CAPN10) had a higher frequency of mutations (Figure 1(f)). In addition, analysis showed significant differences in the CNV of OS-related genes including CLN3, TSC2, DAPK2, LAMP1, ATG16L2, FADD, IKBKB, RAB24, CAPN10, CFLAR, PEX14, MBTPS2, ST13, MAP2K7, and STK11 (Figure 1(g)). Furthermore, LASSO analysis was used to exclude genes that could cause overfitting of the model and to reduce variables (Figures 1(h) and 1(i)). A multivariate Cox regression model was employed to establish a predictive model containing 6 characteristic genes (HEY1, IFNA13, NKX2-3, NR2F1, POU5F1, and YY1) correlated with the prognosis of breast cancer (Figure 1(e)). A BCPRS model was constructed based on the 6 genes. The risk scores were calculated as follows: risk score = 0.3501∗HEY1 + 0.2299∗IFNA + 0.0735∗NKX2 − 3 + 0.1789∗NR2F1 − 0.2976∗POU5F1 − 1.574∗YY1 and BCPRS = log(riskScore).

### 3.2. Evaluation of BCPRS as well as Overall Survival and Clinical Phenotype

**Figure 2:**
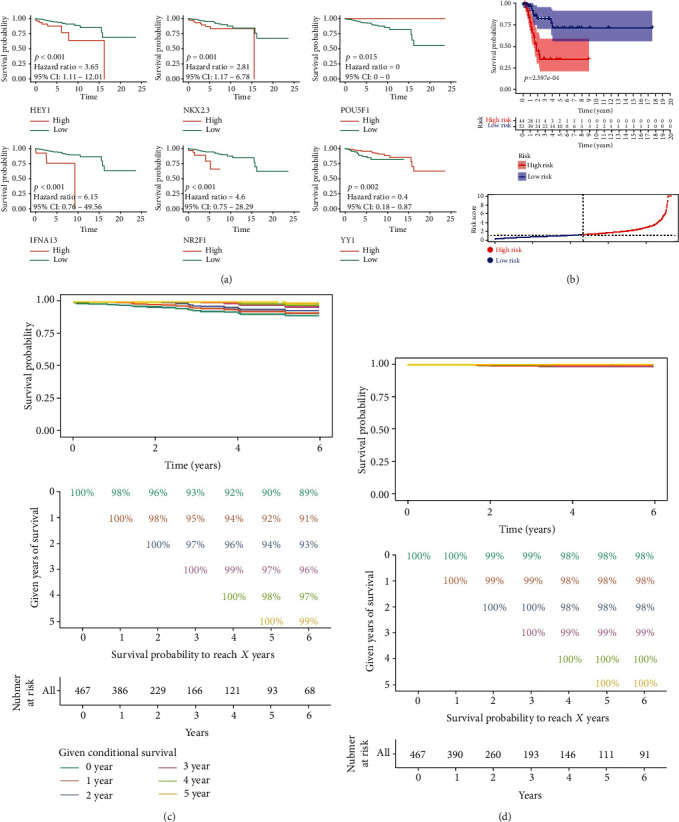
Evaluation of BCPRS and overall survival and clinical phenotypes. (a, b) K-M curves of BCPRS-associated genes selected using the LASSO method (a); A K-M curve of overall survival in each BCPRS group based on the TCGA-BRCA dataset (b). (c, d) Estimated survival rates of patients given a 0–5-year survival period in low/high BCPRS groups. Each column represents the years of survival, and each row represents the percentage of attaining a certain total survival time from the point of survived years.

The Kaplan-Meier (K-M) curve showed that the 6 IMAAGs identified in the previous section were related to the prognosis of breast cancer with good risk prediction capabilities (Figure 2(a)). The low expression level of POU5F1 and YY1 and high expression level of HEY1, IFNA13, NKX2-3, and NR2F1 were significantly related to poor prognosis in breast cancer. Notably, the tumor groups showed a low expression level of HEY1 and NR2F1 compared with the normal group (Supplementary Figure 2E). This implies that HEY1 and NR2F1 may be correlated with a malignant tumor progression phenotype rather than a tumorigenesis phenotype. The K-M curve showed that the risk of death in the high BCPRS group was significantly higher compared with that in the low BCPRS group in the TCGA cohort (Figure 2(b); *p* < 0.001).

The 5-year survival rate of the low-risk group ranged from 98% to 99% and then 100% (1 year, 3 years, and 4 years, respectively). The 5-year survival rate of the low-risk group was better compared with that of the high-risk group (from 89% to 96%) (Figures 2(c) and (d)). Notably, the survival rate of patients in the low-risk group was approximately 100% after 3 years of treatment. This implies that BCPRS could effectively predict the risk of death and recurrence of cancer in breast cancer patients. In addition, the model can help ease the fear of possible recurrence in breast cancer patients in the low-risk group after three years of treatment. Further, it can help ensure a more active follow-up in the high-risk group and in guiding a more reasonable allocation of medical resources.

TNM staging shows severity of a tumor and is used for predicting the prognosis of patients in clinical practice. Interestingly, the findings of this study showed no significant correlation between BCPRS and TNM staging (Supplementary Figure 2A-2D). This implies that BCPRS is independent of tumor staging and can be used as an alternative indicator of tumor prognosis.

### 3.3. Evaluation of the Tumor Immune Microenvironment and Association with BCPRS

**Figure 3:**
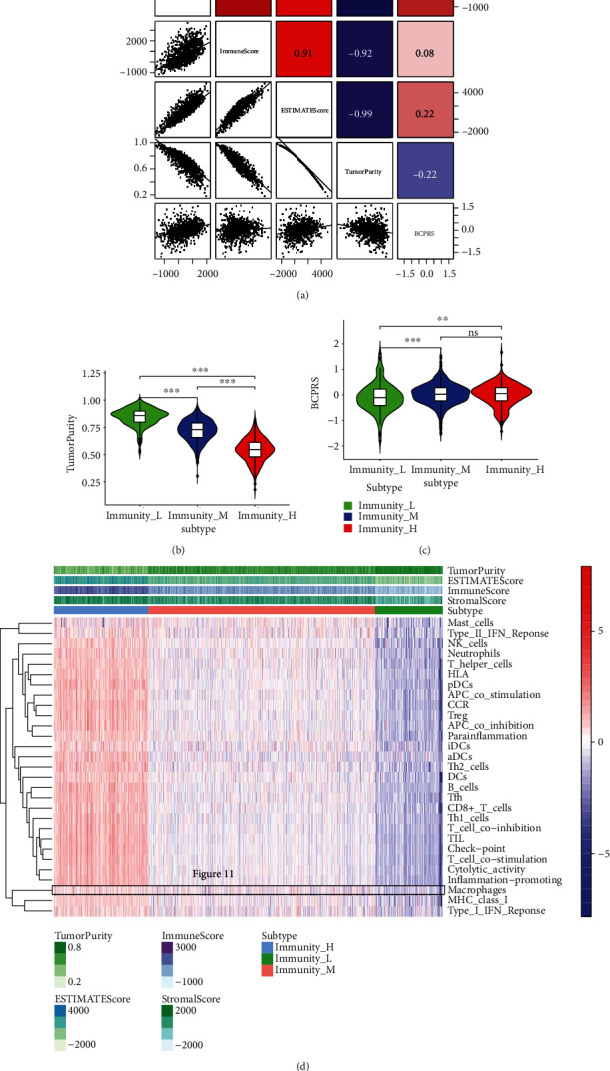
Evaluation of the tumor immune microenvironment and correlation analysis based on BCPRS. (a) Correlations among StromalScore, ImmuneScore, ESTIMATEScore, TumorPurity, and BCPRS in the TCGA-BRCA cohort (*n* = 934). (b, c) Violin figures showing that the immune type in BRCA had a significant correlation with tumor purity and BCPRS (*p* < 0.001). (d) Heat map plots of immune-related functions from the TCGA-BRCA cohort calculated by ssGSEA. Macrophages were highly enriched in the high BCPRS group. The rows represent the gene sets of the samples and the *z*-score values of ssGSEA.

Analysis showed that tumor purity is significantly negatively correlated with ImmuneScore, StromalScore, ESTIMATEScore, and BCPRS (Spearman's correlation, rho = −0.92, -0.82, -0.99, and -0.22, respectively; Figure 3(a)). To further explore this correlation, ssGSEA was used to predict the abundance of immune cells in each sample. Moreover, unsupervised cluster analysis was performed to classify patients into different immune subtypes. The findings showed that tumors with low immune infiltrating subtypes in the TCGA-BRCA cohort had higher purity and lower BCPRS scores compared with those with high immune infiltrating subtypes (Figures 3(b) and 3(c)). These findings indicate that the BCPRS score is highly correlated with specific tumor microenvironment characteristics (such as tumor purity and tumor tissue immune infiltration). A heat map was then constructed to visualize the features (Figure 3(d)).

### 3.4. Differences in the SNPs of Tumor Cells from Different BCPRS Subtypes

The Maftools package was used to explore differences in the distribution of somatic mutations between the low and high BCPRS scores in the TCGA-BRCA cohort. The low BCPRS score group showed a severe burden of tumor mutations compared with the high BCPRS score group. Incidence of the top ten most significant mutation genes was 14.3% versus 12.1%, respectively (Supplementary Figure 3A-3B). Analysis showed that tumor mutations in patients with a high TMB status were correlated with a long-lasting clinical response to immunotherapy. Therefore, we guess that differences in tumor BCPRS scores may mediate clinical response to immunotherapy.

### 3.5. Enrichment Analysis of BCPRS Subtypes

**Figure 4:**
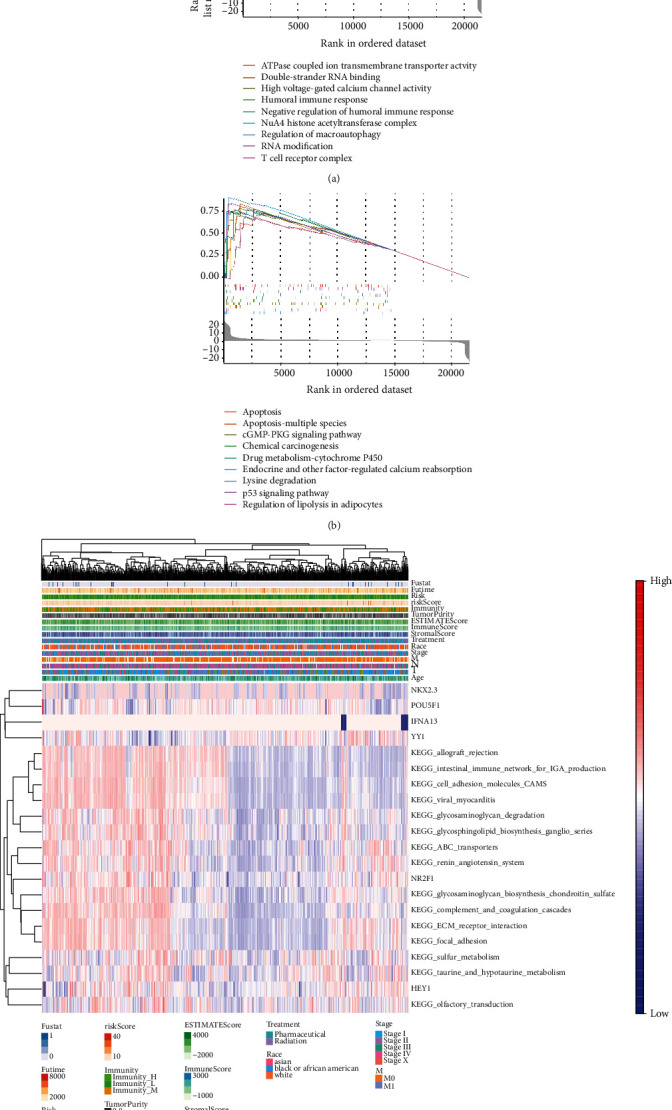
GSVA and GSEA enrichment analyses of BCPRS subtypes. (a, b) GSEA analysis showing significant biological pathways and processes associated with BCPRS (*p* < 0.05): (a) GO enrichment analysis; (b) KEGG enrichment analysis. (c) Heat map plots of BCPRS component genes (IKBKB, ATG16L2, CLN3, MBTPS2, TSC2, and CAPN10) and enrichment analysis by GSVA. Note: Fustat: survival status; Futime: follow-up time.

GO function enrichment analysis was used to explore associated functions of BCPRS. The highly enriched functions included ATPase coupled ion transmembrane transporter activity, double-stranded RNA binding, high voltage-gate calcium channel activity, humoral immune response, negative regulation of humoral immune response, NuA4 histone acetyltransferase complex, regulation of macroautophagy, RNA modification, and T cell receptor complex (Figure 4(a)). In addition, KEGG pathways associated with BCPRS were explored. Highly enriched pathways included apoptosis, cGMP-PKG signaling pathway, chemical carcinogenesis, drug metabolism-cytochrome P450, endocrine and other factor-regulated calcium reabsorption, fatty acid degradation, lysine degradation, p53 signaling pathway, and regulation of lipolysis in adipocytes (Figure 4(b)). These findings show that BCPRS may be associated with the immune, methylation, and autophagy pathways. In addition, BCPRS can indirectly indicate the overall biological function of tumor tissue.

Heat maps based on GSVA analysis and quantification were used to visualize expression of the six key genes and the differentially enriched KEGG pathways (Figure 4(c)). Findings from cluster analysis showed that expression of NR2F1 was significantly correlated with the renin angiotensin system, glycosaminoglycan biosynthesis, chondroitin sulfate, complement and coagulation cascades, and ECM receptor interaction.

### 3.6. Demographic, Clinicopathological, and Tumor Microenvironment Characteristics of BRCA Patients in High and Low BCPRS Groups

Demographic, clinicopathological, and tumor microenvironmental characteristics of patients with high and low BCPRS/BCRRS are presented in Tables 1 and 2. Analysis showed that the low and high BCPRS groups were significantly heterogeneous in terms of clinicopathological and tumor microenvironment characteristic factors (immunity groups, StromalScore, ImmuneScore, ESTIMATEScore, TumorPurity, and mRNAsi; Table 1). The high BCPRS group showed higher immune scores with lower tumor purity. Notably, mRNAsi was lower in the high BCPRS group compared with the low BCPRS group, implying that the BCPRS score is negatively correlated with breast cancer cell stemness. The findings of this study were consistent with findings from previous studies that the BCRRS score is significantly correlated with malignancy of breast cancer (Table 2). This indicates that BCPRS is a prognostic factor independent of cancer cell stemness characteristics. The follow-up time in the low-risk group was longer compared with that in the high-risk group (*p* < 0.005), indicating that the results were valid over time. Age, race, and treatment did not differ significantly across BCPRS groups implying that BCPRS is a potential prognostic risk factor for breast cancer independent of age, race, and treatment. To further validate these findings, multifactorial COX analysis was performed which showed that BCPRS (*p* < 0.001) an independent risk factor for BRCA prognosis with better predictive power compared with other tumor microenvironmental features (immunity groups, StromalScore, ImmuneScore, ESTIMATEScore, TumorPurity, and mRNAsi) (Table 3). In summary, BCPRS is correlated with several tumor microenvironmental features and is a prognostic factor independent of tumor cell stemness scores (mRNAsi) and clinical TNM stage pathology.

### 3.7. Construction and Verification of a Breast Cancer OS Nomogram Prediction Model

**Figure 5:**
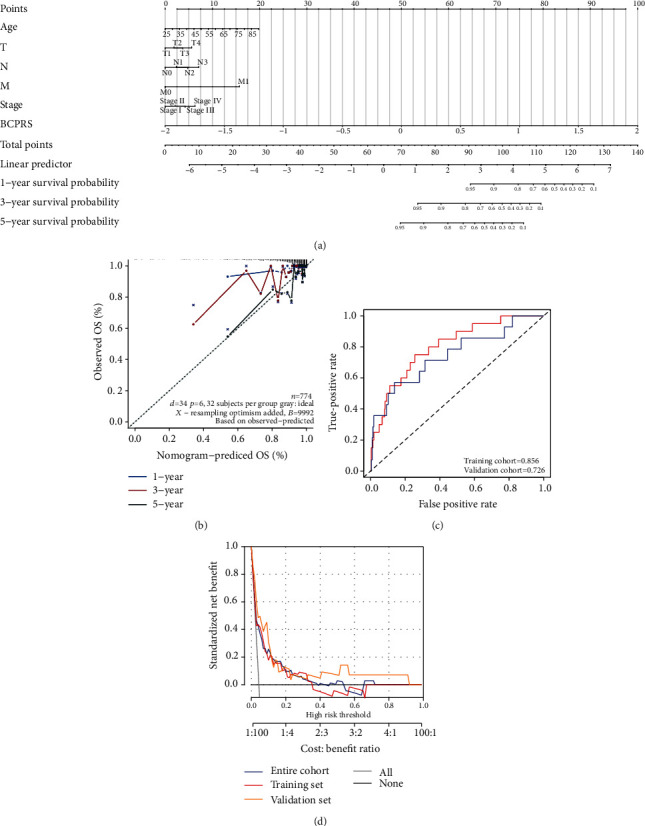
Construction and verification of a breast cancer OS nomogram prediction model. (a) A nomogram prediction model for the prognosis of OS in breast cancer. Age, T, N, M, stage, and BCPRS were included. (b) Plots showing the calibration of nomograms based on the breast cancer OS nomogram prediction model. (c) ROC analysis showing the predictive ability of the breast cancer OS nomogram model based on the TCGA-BRCA cohort and validated by the clinical cohort. (d) Decision curve analyses of the breast cancer OS nomogram model based on the TCGA-BRCA cohort and validated by the clinical cohort.

Characteristics of the tumor microenvironment such as immunity groups, StromalScore, ImmuneScore, ESTIMATEScore, TumorPurity, and mRNAsi are often difficult to obtain in clinical work. Therefore, to develop a more applicable prediction model, a BRCS OS nomogram prediction model was constructed based on age, T, N, M, stage, and BCPRS. The OS nomogram prediction model for breast cancer included BCPRS and clinicopathological parameters as shown in Figure 5(a) and Supplementary Table 2. The calibration curve showed that the OS nomogram was highly accurate in predicting the 5-year survival rate compared with the ideal model (Figure 5(b)). The area under the ROC curve (AUC) of the training cohort was 0.856 whereas the AUC of the validation cohort was 0.726 (Figure 5(c)). Moreover, the training group C-index of the breast cancer OS nomogram prediction model was higher (0.802, 95% CI, 0.709-0.895) compared with that of the validation cohort (0.747, 95% CI, 0.600-0.894; Table 4). In addition, it was higher compared with that of the entire cohort which was 0.767 (95% CI, 0.681-0.853), indicating that the model had a good predictive power for the prognosis of breast cancer. Furthermore, decision curve analysis (DCA) of the nomogram showed that the prediction model had good clinical value (Figure 5(d)).

### 3.8. Construction and Verification of the Breast Cancer PFS Nomogram Prediction Model Based on the Clinical Cohort

**Figure 6:**
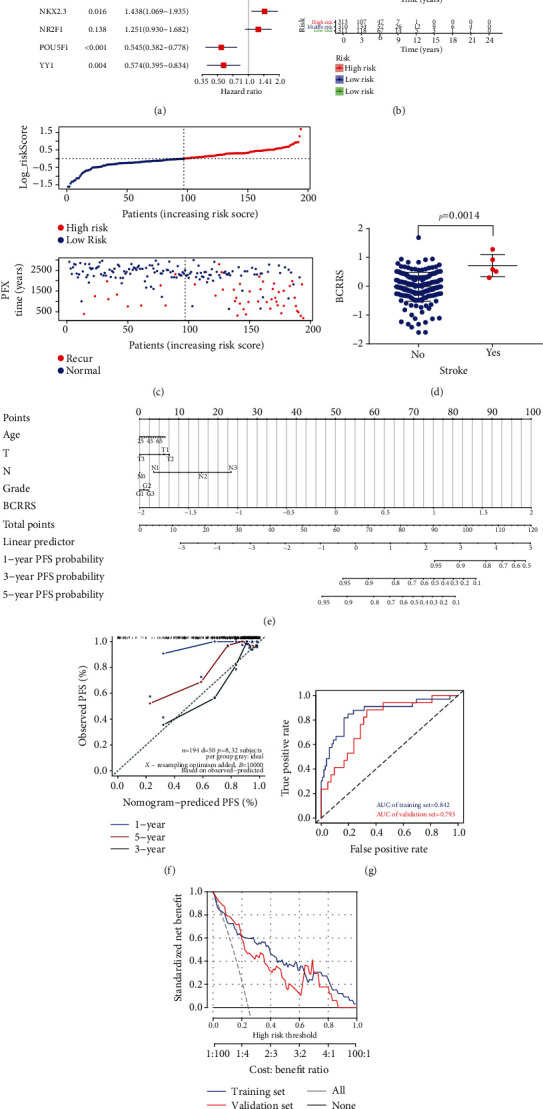
Construction and verification of a breast cancer PFS nomogram prediction model based on the clinical cohort. (a) Forest plot of multivariate Cox regression analysis showing the PFS-related values of BCRRS. (b) K-M curves of PFS survival as per BCRRS groups in the clinical cohort. (c) Distribution of BCRRS in the clinical cohort. Top panel: classification of patients into different groups based on the BCRRS scores. Bottom panel: distribution of patients' status and PFS time. (d) Relative level of BCRRS in patients with and without stroke history after breast cancer. Significant differences were observed (*p* = 0.0014). (e) A nomogram prediction model for the prognosis of PFS in breast cancer. Age, T, N, grade, and log_riskScore (BCRRS) were included. (f) Plots showing the calibration of nomograms based on the breast cancer OS nomogram prediction model. (g) ROC analysis was used to validate the predictive ability of the breast cancer PFS nomogram model based on the clinical cohort. (h) Decision curve analyses of the breast cancer PFS nomogram model based on the clinical cohort.

Basic clinical data for 194 breast cancer patients was obtained from the Shanghai First People's Hospital, and the 6-gene signature was analyzed by PCR (Table 2). Expression levels of the genes were normalized using the *z*-scoring process. The 6-gene signature was then used for multifactor COX regression analyses (Figure 6(a)) (Supplementary Table 3). BCRRS is calculated following a similar method as described for BCPRS. The difference only is that the BCRRS uses clinical data to predict risk of breast cancer recurrence, whereas BCPRS uses TCGA data to predict risk of death in breast cancer patients. The Kaplan-Meier curve analysis showed that the PFS of the low BCRRS group was significantly higher compared with that of the pRS group (*p* < 0.001; Figure 6(b)). The 6-gene signature was then curated to construct the Breast Cancer Recurrence Risk Score (BCRRS) system (Figure 6(c)). The findings showed that BCRRS was associated with risk of stroke (Figure 6(d)).

A PFS nomogram model for breast cancer patients was constructed by combining the clinical characteristics of tumor patients and BCRRS scores, to determine the risk of tumor recurrence (Figure 6(e)). The calibration curve indicated that the PFS nomogram had higher predictive value compared with the ideal model (Figure 6(f)). The AUC of the model in the training cohort was 0.842 whereas that of the validation cohort was 0.726 (Figure 6(g)). The C-index of the breast cancer PFS nomogram prediction model for the training group was higher (0.864, 95% CI, 0.784-0.944) compared with that of the validation cohort, 0.793 (95% CI, 0.672-0.914), and the entire cohort, 0.843 (95% CI, 0.776-0.909) (Table 4). These findings indicate that the model had a good predictive value for the prognosis of breast cancer. Analysis of the DCA curve of the breast cancer PFS nomogram prediction model further showed that the prediction model had good clinical value (Figure 6(h)).

### 3.9. WGCNA Shows Module Relationships

**Figure 7:**
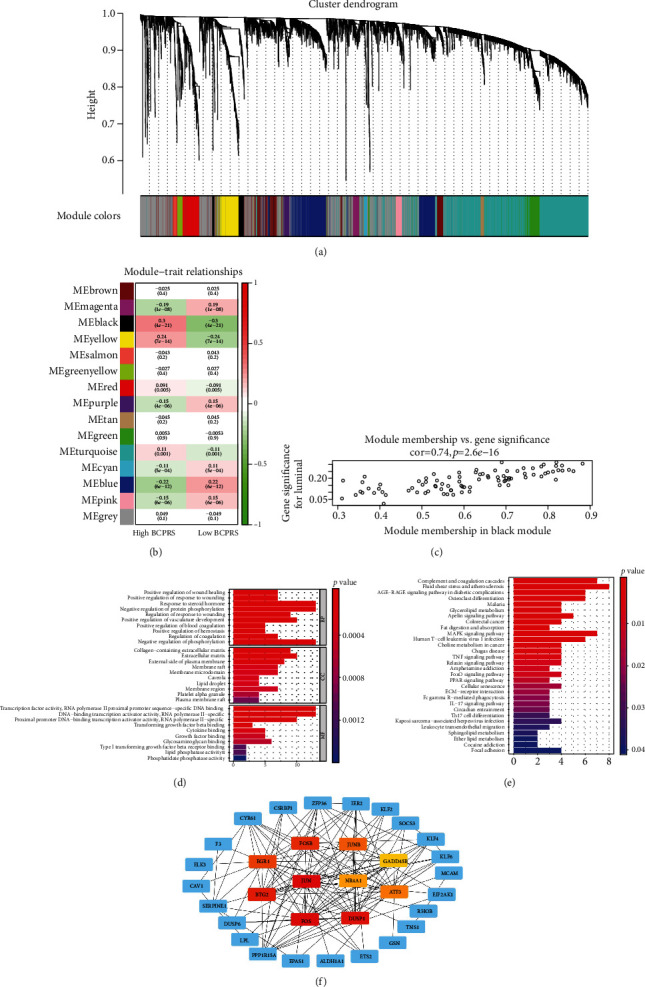
WGCNA-related analysis based on BCPRS groups. (a) Identification of weighted gene coexpression network modules in the TCGA-BRCA dataset. (b) A heat map of the correlation between module eigengenes and the BCPRS phenotype in breast cancer. (c) Correlation analysis of black module gene members and gene significance (cor = 0.74, *p* < 0.001). (d, e) GO and KEGG enrichment analyses of black module genes: (d) GO enrichment analysis; (e) KEGG pathway analysis. Note: *X*-axis label represents the FDR. (f) Protein-protein interaction (PPI) network of genes from the black module. Red represents a strong correlation. FOSB, JUNB, EGR1, GADD45B, JUN, NR4A1, BTG2, ATF3, FOS, and DUSP1 were used as the hub genes of this network.

Genes selected from the 936 breast cancer patient samples from the TCGA-BRCA database were used for WGCNA network construction. The maximum expression difference threshold was 25%. In this study, high-scale independence (near 0.9) and low average connectivity (near 0) were attained by setting the power at 5. The combined threshold was set at 0.25 and 15 modules and was represented as various colors (Figure 7(a)). The black module (cor = 0.3, *p* < 0.0001) represented genes positively correlated with BCPRS, whereas the blue module (cor = −0.22, *p* < 0.0001) represented genes negatively associated with BCPRS (Figure 7(b)). A total of 87 genes were grouped into the black module. Analysis showed that the genes in the black module were highly correlated with their corresponding module and were significantly correlated with the traits of BCPRS (Figure 7(c)). This finding shows that the genes in the black module should be explored further.

### 3.10. GO Function and KEGG Pathway Enrichment Analyses of Core Genes

GO function (BP, CC, and MF) and KEGG pathway enrichment analyses of the core genes in the black module were performed. These core genes were implicated in several biological processes including positive regulation of wound healing, positive regulation of response to wounding, response to steroid hormone, negative regulation of protein phosphorylation, regulation of response to wounding, positive regulation of vasculature development, positive regulation of blood coagulation, positive regulation of hemostasis, regulation of coagulation, and negative regulation of phosphorylation (Figure 7(d)). In addition, the core genes were associated with cellular components including collagen-containing extracellular matrix, extracellular matrix, external side of plasma membrane, membrane raft, membrane microdomain, caveola, lipid droplet, membrane region, platelet alpha granule, and plasma membrane raft. Further, the core genes were implicated in various molecular functions including transcription factor activity, RNA polymerase II proximal promoter sequence-specific DNA binding, DNA-binding transcription activator activity, RNA polymerase II-specific, transforming growth factor beta binding, cytokine binding, growth factor binding, glycosaminoglycan binding, type I transforming growth factor beta receptor binding, lipid phosphatase activity, and phosphatidate phosphatase activity.

In addition, KEGG pathway analysis showed that these genes were mainly involved in complement and coagulation cascades, fluid shear stress and atherosclerosis, AGE-RAGE signaling pathway in diabetic complications, osteoclast differentiation, malaria, glycerolipid metabolism, apelin signaling pathway, colorectal cancer, fat digestion and absorption, MAPK signaling pathway, human T-cell leukemia virus 1 infection, choline metabolism in cancer, Chagas disease, and TNF signaling pathway (*p* < 0.05; Figure 7(e)). KEGG and GO enrichment analyses showed that the black module genes correlated with BCPRS may be involved in tumor proliferation, invasion, and metastasis. In addition, these genes may be the key genes implicated in the poor prognosis of breast cancer.

### 3.11. Construction and Validation of the PPI Network Based on WGCNA Analysis

A total of 86 nodes and 266 edges were identified in the core genes of the black module using the STRING database with a PPI enrichment *p* value of 1.0*e*-16 (Supplementary Figure 4A). Modules were identified, and 33 hub genes screened using the PPI network (Figure 7(f)). Notably, EGR1, BTG2, FOSB, JUN, FOS, JUNB, NR4A1, DUSP1, GADD45B, and ATF1 played important roles in the network. The overall survival data showed that the prognosis of breast cancer patients was poorer when two genes (FOS and FOSB) were highly expressed or when four genes (JUN, GADD45B, NR4A1, and BTG2) showed low expression levels (Supplementary Figure 4B). The findings showed that BCPRS was correlated with the expression of JUNB, DUSP1, JUN, FOS, EGR1, FOSB, ATF1, GADD45B, NR4A1, and BTG2 (Spearman's correlation, rho = 0.19, 0.26, 0.12, 0.17, 0.16, 0.2, −0.08, 0.18, 0.12, and 0.21, respectively; Supplementary Figure 4C). These findings show that BCPRS can be used to effectively identify genes and related pathways correlated with the high risk of poor prognosis in breast cancer.

### 3.12. Construction of a Drug-ceRNA Network Based on BCPRS

All BCPRS-related lncRNAs, miRNAs, and mRNAs were retrieved using the R software. A drug-lncRNA network was then constructed based on the prediction results of the LncMAP database (Supplementary Figure 5). The relationship between BCPRS-related lncRNAs, BCPRS-related miRNAs, and BCPRS-related mRNAs was explored using miRTarBase and miRWalk databases (Supplementary Figure 5). A potential regulatory drug-ceRNA network was then constructed.

### 3.13. Identification of Cell Clusters in Human TNBC Cells Shows High Cell Heterogeneity

**Figure 8:**
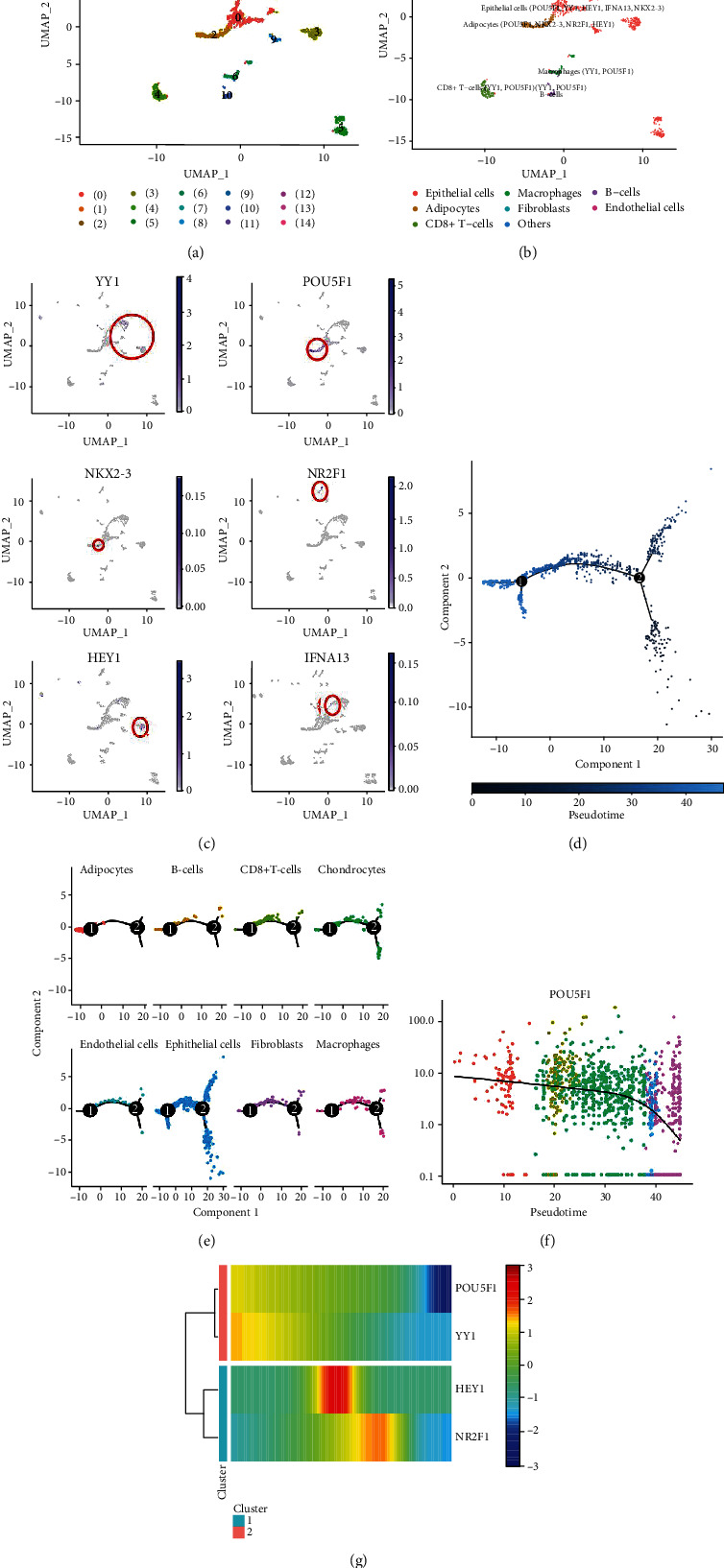
Cell annotation and trajectory analysis of TNBC cell subsets. (a, b) All 14 clusters of cells in TNBC were annotated by singleR and CellMarker. (c) Expression of six BCPRS-related genes (YY1, POU5F1, NKX2-3, NR2F1, HEY1, and IFNA13) in scRNA-seq is shown. Blue represents high expression and gray represents low expression level. (d) Trajectory analysis showed that TNBC cells had distinct differentiation patterns. (e) TNBC adipocyte cells were mainly located in the root, whereas epithelial cells, macrophages, and others were located in either branch or root. (f, g) Trajectory analysis showed the differential expression of genes (POU5F1, YY1, HEY1, and NR2F1) at different pseudotimes.

A total of 1266 cells were included for analysis after quality control of 1535 cells in the tumor core of six human TNBC samples (Supplementary Figure 6A). ANOVA plots showed 1783 corresponding genes in all TNBC cells, and the top 20 marker genes for each cell cluster were labeled (Supplementary Figure 6B). The number of detected genes was significantly correlated with the sequencing depth, with a Pearson correlation coefficient of 0.63 (Supplementary Figure 6C). Principal component analysis (PCA) showed no significant separation of these TNBC cells, and 20 PCs were identified (estimated *p* value < 0.05; Supplementary Figure 6D-6F). The Uniform Manifold Approximation and Projection (UMAP) algorithm was used to accurately group human TNBC cells into 14 individual clusters (Figure 8(a)). The top 20 marker genes for each cell cluster and clustering of different cell clusters were identified (Supplementary Figure 6G and 6H). The clusters were then annotated with singleR and CellMarker tools based on the expression pattern of the marker genes (Figure 8(b)). Expression of six BCPRS-related genes (YY1, POU5F1, NKX2-3, NR2F1, HEY1, and IFNA13) was determined using scRNA-seq (Figure 8(c) and Supplementary Figure 7A). Tabula Muris is a compendium of single cell transcriptome data from the model organism Mus musculus, containing nearly 100,000 cells from 20 organs and tissues [74]. Expression of the six BCPRS genes (IFNA13, HEY1, NKX2-3, NR2F1, POU5F1, and YY1) in breast cancer tissues was analyzed using Tabula Muris's FACS and droplet methods (Supplementary Figure 8). NKX2-3 and IFNA13 showed low expression levels in normal breast tissues, however, were highly expressed in breast cancer tissues. POU5F1 was mainly expressed in adipocytes.

Trajectory analysis was used to explore TNBC cells with distinct differentiation patterns (Figure 8(d)). TNBC adipocytes cells were mainly located in the root (a much early pseudotime), whereas epithelial cells and macrophages and others were located in either the branch or root (Figure 8(e)). Furthermore, trajectory analysis showed differential expression of genes (POU5F1, YY1, HEY1, and NR2F1) at different pseudotimes (Figures 8(e)–8(f)).

### 3.14. Adipocytes and Adipose Tissue Macrophages (ATMs) Are Enriched in High BCPRS Cluster

**Figure 9:**
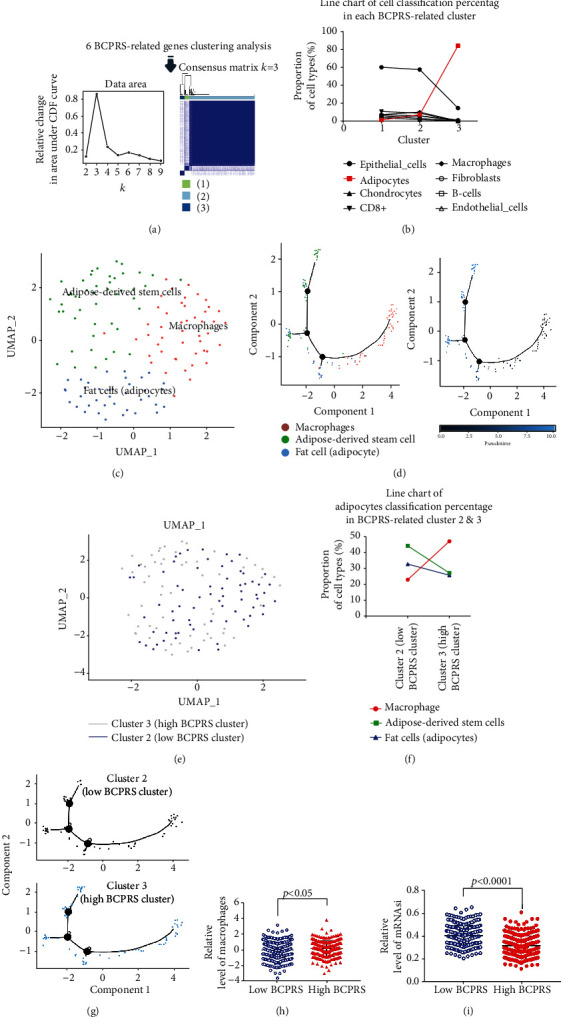
Clustering analysis of six BCPRS-related genes and cell annotation of TNBC adipocyte subsets. (a) Six-clustering analysis of BCPRS-related genes groups TNBC cells into three clusters; cluster 3 was defined as low BCPRS whereas cluster 2 was defined as the high BCPRS group. (b) Line chart of cell classification percentage in each BCPRS-related cluster. (c) All 3 clusters of adipocytes in TNBC were annotated by CellMarker. (d) Trajectory analysis showed differential distribution of cells (macrophages, adipose-derived stem cells, and fat cells) at different pseudotimes. (e) Distribution of cluster 2 (low BCPRS cluster) and cluster 1 (high BCPRS cluster) in adipocytes. (f, g) Line chart of adipocyte percentage in BCPRS-related clusters 2 and 3 (f); trajectory analysis showed the differential distribution of high/low BCPRS cluster at different pseudotimes (g). (h) Relative level of macrophages in low and high BCPRS groups. Significant differences were observed (*p* < 0.0001). (i) Relative level of miRNAsi in low and high BCPRS groups. Significant differences were observed (*p* < 0.0001).

Clustering analysis of six BCPRS-related genes (IMAAGs) grouped cells into three clusters (Figure 9(a)). Cluster 3 was defined as a low BCPRS cluster whereas cluster 2 was defined as a high BCPRS group. Notably, adipocytes were mainly located in cluster 3 (Figure 9(b) and Supplementary Figure 7B). This finding indicates that a high degree of adipocyte infiltration may be correlated with the poor prognosis of high BCPRS in breast cancer. The UMAP algorithm was used to successfully group human TNBC adipocytes into 3 individual clusters. The clusters were then annotated with the CellMarker tool based on the expression pattern of the marker genes (Figure 9(c)). Trajectory analysis showed that TNBC adipocytes had distinct differentiation patterns (Figure 9(d) and Supplementary Figure 9A, 9B). Analysis only showed a few cells of cluster 1 in the adipose tissue, and the difference of DEGs between clusters 2 and 3 was highly significant. Adipocytes were mainly located in cluster 2 (Figures 9(e) and 9(f)). Adipose tissue macrophages (ATMs) (CD68+) were highly enriched in high BRPRS clusters (*p* < 0.05) (Figures 9(f)–9(h)). Analysis showed that macrophages were highly enriched in the high BCPRS group, whereas the relative level of mRNAsi was lower in the high BCPRS group compared with the lower BCPRS group (Figure 9(i)). Therefore, high infiltration of ATMs (CD68+) may be correlated with induction of BCPRS upregulation. High BCPRS characteristics may present malignant characteristics different from the stem cellularity of BRCA cells.

### 3.15. Ligand-Receptor Interaction Analysis and Identification of Hub Genes

**Figure 10:**
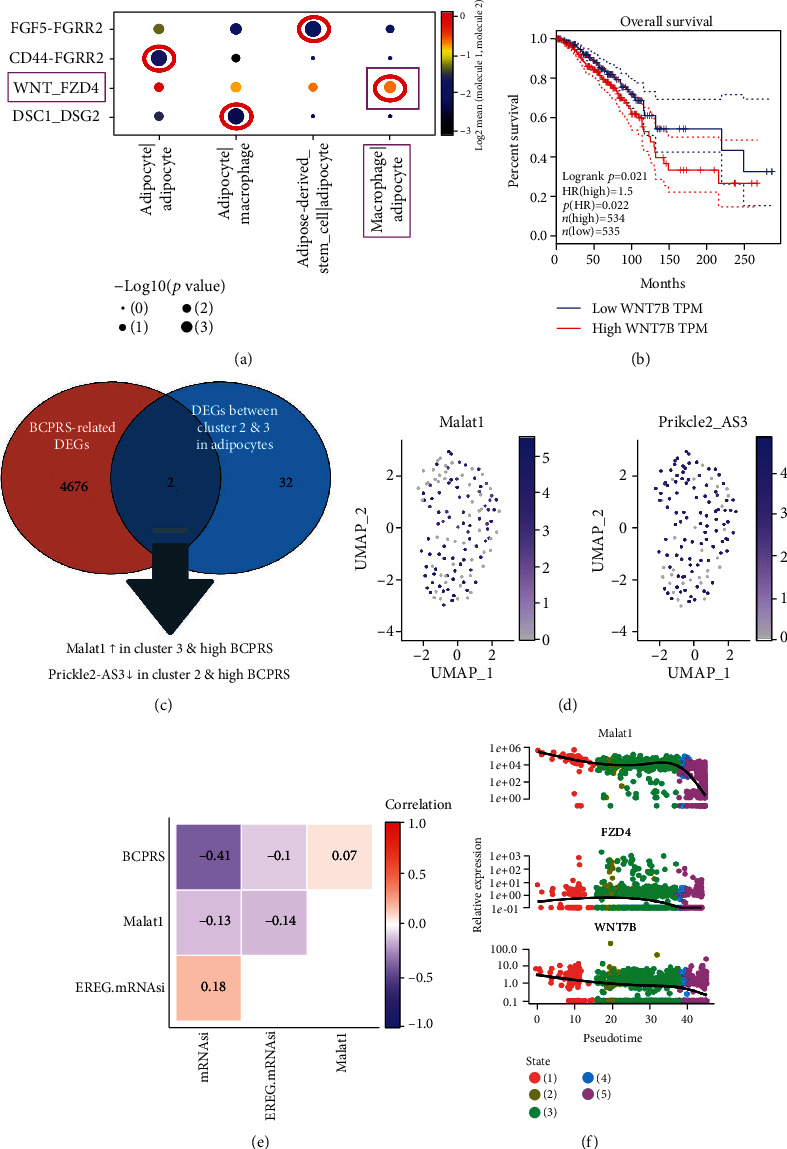
Ligand-receptor interaction analysis and identification of hub genes. (a) Receptor-ligand interaction within each subtype of each cluster of adipocytes. (b) K-M curves for Wnt7b in the TCGA BRCA cohort. (c) Venn diagram showing intersection of genes in BCPRS-related DEGs and DEGs between clusters 2 & 3 in adipocytes. (d) Expression levels of MALAT1 and PRICKLE-AS3 in scRNA-seq from TNBC adipocytes. Blue represents high expression level and gray represents low expression level. (e) Correlations among BCPRS, MALAT1, EREG.mRNAsi, and mRNAsi in BRCA tissues (TCGA cohort). (f) Trajectory analysis showing the differential expression of genes (MALAT1, FZD4, and Wnt7b) at different pseudotimes.

CellPhoneDB was used to infer cell-to-cell communication to explore differences and commonalities between each subtype in the information exchange. Receptor-ligand interactions within each subtype of each cluster of adipocytes were analyzed (Figure 10(a)). A high expression of Wnt7b was associated with poor breast cancer prognosis (Figure 10(b)). This implies that the interaction of Wnt7b and FZD4 between ATMs (CD68+) and adipocytes (FCs) may contribute to the poor prognosis of breast cancer, mainly in the form of a high BCPRS profile.

Hub genes between ATMs (CD68+) and adipocytes (FCs) were identified by getting the intersection in BCPRS-related DEGs. A Venn diagram was constructed to show the intersection of genes in BCPRS-related DEGs and DEGs between clusters 2 and 3 in adipocytes. MALAT1 and PRICKLE2-AS3 were defined as common Differentially Expressed Genes (Figure 10(c)). MALAT1 was highly expressed in cluster 3 and in the high BCPRS group. Expression of MALAT1 and PRICKLE-AS3 using scRNA-seq of TNBC adipocytes is presented in Figure 10(d) and Supplementary Figure 9C. Correlation analysis showed that the expression level of MALAT1 was significantly correlated with the expression of other genes (YY1, POU5F1, NR2F1, IFNA13, and HEY1) in the TCGA BRCA dataset (Supplementary Figure 7C). Similar to BRPRS, the expression level of MALAT1 was negatively correlated with mRNAsi and EREG.mRNAsi (Figure 10(e)). Trajectory analysis showed that MALAT1, FZD4, and Wnt7b were highly expressed in state 1 similar to POU5F1 and adipocytes (Figure 10(f)). Therefore, MALAT1, FZD4, and Wnt7b were defined as hub genes related with BCPRS.

### 3.16. LINC00276&MALAT1/miR-206/FZD4-Wnt7b Pathway Was Predicted

**Figure 11:**
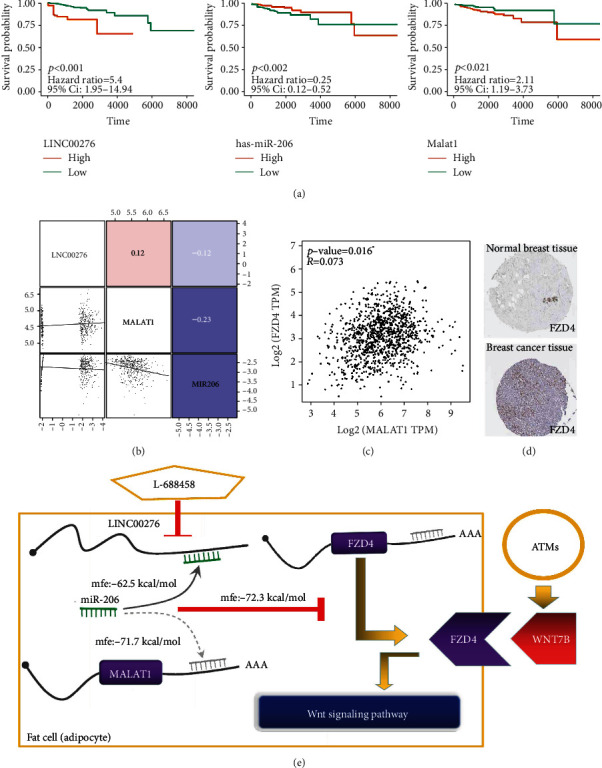
Prediction of LINC00276&MALAT1/miR-206/FZD4-Wnt7b pathway. (a) Survival analysis curve of LINC00276, has-miR-206, and MALAT1. (b) Correlations among LINC00276, miR-206, and MALAT1 in BRCA tissues (TCGA cohort). (c) Correlation analysis showed that expression of MALAT1 and expression of FZD4 were significantly correlated in TCGA BRCA data. (d) Antibody staining immunohistochemistry images of FZD4 in normal and cancer breast tissues obtained from THPA. (e) A model showing prediction of the LINC00276&MALAT1/miR-206/FZD4-Wnt7b pathway.

Survival analysis was performed to identify potential MALAT1-related lncRNAs/miRNAs from BCPRS-related lncRNAs and miRNAs (Figure 11(a) and Supplementary Figure 5). The low expression of miR-206 and high expression of LINC00276 and MALAT1 were significantly correlated with the poor prognosis in breast cancer. Bioinformatics analysis using RNAhybrid 2.12 showed that miR-206 is the potential target miRNA of LINC00276 (mfe: -62.5 kcal/mol), MALAT1 (mfe: -71.7 kcal/mol), and FZD4 (mfe: -72.3 kcal/mol) (Supplementary Figure 9D). Correlation analysis using TCGA data showed that MALAT1 was positively correlated with LINC00276 and FZD4 expression and negatively correlated with miR-206 expression. In addition, previous studies report that MALAT1 can act as a miR-206 sponge (Figures 11(b) and 11(c)) [31, 32, 75]. THPA (https://www.proteinatlas.org/) was used to explore the expression of FZD4 in normal and cancer breast tissues. Analysis showed that FZD4 was highly expressed in breast cancer tissues compared with normal breast tissues (Figure 11(d)). These findings show that MALAT1 and LINC00276 (regulated by L-685458) act as sponges for miR-206, thus promoting FZD4 transcription, and upregulate the Wnt signaling pathway in the presence of Wnt7b secreted by ATMs (CD68+). This process may be interrupted by L-685458 (Figure 11(e)).

### 3.17. Prediction of Breast Cancer Cell Types with BCPRS-Related Gene Signatures

**Figure 12:**
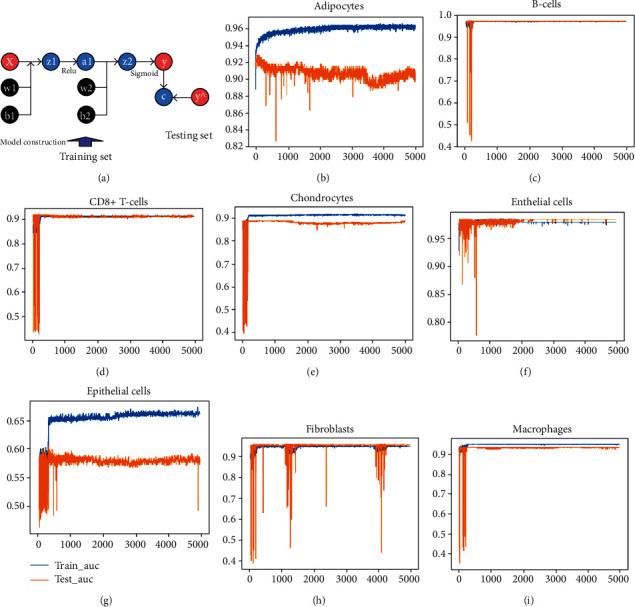
Hub BCPRS-related gene signature for prediction of breast cancer cell types. (a) A schematic diagram of the neural network. (b–h) The ROC plot in the training set and the validation set used to validate the accuracy of the network's prediction capacity.

The BCPRS-related genes (YY1, POU5F1, NKX2-3, NR2F1, HEY1, and IFNA13) showed high heterogeneity in different cells; thus, the genes can independently predict cellular composition to reflect the microenvironment of tumor tissues. Therefore, a neural network-based model was constructed to predict cell types in breast cancer tissues based on genes YY1, POU5F1, NKX2-3, NR2F1, HEY1, and IFNA13 (Figure 12(a)). The area under the curve (AUC) of ROCs was high (Figures 12(b)–12(i)). This finding indicates that these models had good predictive power, especially in predicting adipocytes (AUC ≈ 0.96), fibroblasts (AUC ≈ 0.95), and endothelial cells (AUC ≈ 0.98). This implies that these genes can be used to map the tumor microenvironment.

## 4. Discussion

The current study was conducted based on immune, methylation, and autophagy perspectives. A total of 6 prognostic IMAAGs were screened and identified to comprehensively analyze genes associated with the prognosis of OS and PFS in breast cancer. The findings of this study showed that the BCPRS and BCRRS scoring systems based on 6 IMAAGs accurately stratified the prognosis of breast cancer patients. OS and PFS nomogram prediction models were constructed with satisfactory clinical values. Notably, BCRRS was associated with the risk of stroke. Adipocytes and adipose tissue macrophages (ATMs) were highly enriched in the high BCPRS cluster and were associated with poor prognosis. Ligand-receptor interactions and potential regulatory mechanisms were explored. The LINC00276&MALAT1/miR-206/FZD4-Wnt7b pathway was identified which may be useful in future research on targets against breast cancer metastasis and recurrence. Neural network-based deep learning modes based on the BCPRS-related gene signatures were established and showed high accuracy in cell type prediction.

Overall survival analysis using the BCPRS score showed that the survival rate of patients in the low BCPRS group within 5 years of treatment was as high as 98%. This rate was significantly higher compared with the survival rate in the high BCPRS group (90%). However, after 3 years of treatment, the survival rates in the two groups were almost similar. This finding showed that the CS rate gradually increased as the survival rate of patients in both groups gradually stabilized. Patients prefer individualized prediction of survival probability; therefore, this information may help in coping with the fear of recurrence or death and can be used in the design of personalized follow-up plans [76–78].

Malta et al. reported that mRNAsi can be used to determine stem cell differentiation levels [42]. Previous studies report that T4 and stage IV have a relatively higher mRNAsi value [34], whereas the mRNAsi value was negatively correlated with BCPRS in the current study. In addition, studies report that BCPRS are not significantly correlated with TNM staging, as the TNM stage does not reveal the biological characteristics of the tumor [79]. This implies that the TNM stage is not sufficient in reflecting prognosis and predicting the efficacy of tumor treatment. Therefore, TNM staging should be combined with other predictors to form a comprehensive risk assessment model for breast cancer prognosis [79]. In the current study, BCPRS was a prognosis factor independent of TNM staging. Analysis of the nomogram showed that the predictive ability of BCPRS was superior compared with that of TNM staging alone. Therefore, the findings of the current study show that BCPRS is a predictive factor independent from tumor cell stemness scores (mRNAsi) and clinical TNM stage pathology. A comprehensive evaluation of the BCPRS, mRNAsi, and TNM scoring systems in this study therefore provides beneficial insights on the prognosis of breast cancer.

The findings of this study showed a significant association between IMAAG genes. The six genes used in the BCPRS and BCRRS scoring systems were highly correlated with the prognosis of OS and PFS in breast cancer. Higher BCPRS and BCRRS scores of breast cancer patients were correlated with worse prognosis. Moreover, GSEA and GEVA enrichment analyses showed that the BCPRS score was significantly correlated with the differences in the biological pathways involved in immune infiltration, autophagy, and methylation. Notably, WGCNA analysis showed consistent findings as enrichment analyses. KEGG and GO enrichment analyses of BCPRS-related genes derived from WGCNA analysis indicated that the BCPRS-related genes are involved in tumor proliferation, invasion, and metastasis. Therefore, BCPRS-related genes may significantly contribute to the poor prognosis of breast cancer. In addition, BCPRS can be used to comprehensively determine the status of autophagy, methylation, and immune enrichment, thus enhancing knowledge of the tumor microenvironment in breast cancer cells [6].

The identified IMAAGs are potential prognosis factors of OS and PFS in breast cancer patients. Notably, most of these genes have been reported in previous studies to be closely correlated with the prognosis of cancer patients, including breast cancer. BRCA1 is the key molecule in breast cancer and is mainly expressed at low levels in breast cancer. Its expression is positively correlated with that of YY1. Previous studies report that YY1 binds to the BRCA1 promoter, and overexpression of YY1 leads to increased expression of BRCA and several genes downstream of BRCA1 [80]. In addition, several studies report that a high expression of YY1 may be beneficial in the prognosis of breast cancer [81–83]. Borgen et al. reported that NR2F1 is a potential disseminated tumor cell arousal factor that promotes bone metastasis in breast cancer [84]. Moreover, NKX2-3 modulates the development of colorectal cancer by regulating the Wnt signaling pathway [85]. NKX2-3 can also be used as a diagnostic marker for prostate cancer [86]. Previous studies report that IFNA13 may be a potential molecular marker for the prognosis of colon cancer [87]. In addition, HEY1-related pathways modulate the cellular plasticity of liver cancer tumors, which is one of the risk factors for the disease [88]. Furthermore, the NOTCH4-HEY1 pathway induces epithelial-mesenchymal transition in head and neck squamous cell carcinoma [89]. POU5F1 plays an important role in lung and colon cancers [90–92]. In breast cancer, POU5F1 is associated with the ER*α*'s tumor suppressor function [93].

In the current study, adipocytes were mainly located in high BCPRS clusters. Studies report that adipocytes have a complex function in BRCA [94–97]. Previous studies report that adipose tissue macrophages may accumulate in the mammary adipose tissue as a mechanism for promoting TNBC stemness and tumorigenesis during obesity [98]. Notably, adipose tissue macrophages (ATMs) were enriched in high BCPRS clusters in the current study. This finding indicates that macrophages do not always play a role in promoting the health of the organism and are sometimes responsible for the malignant transformation of the tumor in breast cancer consistent with previous studies [99]. However, further studies should explore the initial bidirectional signaling between BRCA microenvironment cell signaling and adipocytes [100]. The findings imply that cells that clustered together were from the same anatomical region and the same clonal expansion [101].

The findings also showed that Wnt7b secreted by ATMs may activate the Wnt signaling pathway in the tumor immune microenvironment through interactions with FZD4, ultimately causing malignant transformation of breast cancer. Studies report that upregulation of Wnt7b is necessary for invasion, growth, and metastasis of BRCA through activation of the Wnt signaling pathway [102, 103]. FZD4 acts as a receptor for Wnt7b and plays an essential role in the activation of the Wnt signaling pathway [104]. Wnt signaling is important in stem cell biology and regenerative medicine. Bioinformatics and correlation analysis showed that mRNA of FZD4 has a strong minimal free energy with miR-206, and transcription of FZD4 in adipocytes may be inhibited by miR-206. Previous studies report that MALAT1 can act as a miR-206 sponge [31, 32, 75]. MALAT1 induces cancer cell proliferation, invasion, and migration in mice [105]. However, oncogenic and tumor-suppressive functions of MALAT1 in breast cancer cells are controversial [105]. Similar to BRPRS, the expression level of MALAT1 was negatively correlated with mRNAsi and EREG.mRNAsi. This finding implies that MALAT1 may be a double-edged sword whose oncogenic effects may be correlated with the BCPRS-associated tumor microenvironment, which is negatively correlated with tumor cell stemness. The findings of the current study showed that LINC00276 acts as a miR-206 sponge to upregulate FZD4 transcription. MALAT1 and LINC00276 (regulated by L-685458) thus act synergistically as sponges for miR-206, which in turn promotes FZD4 transcription and upregulates the Wnt signaling pathway in the presence of Wnt7b secreted by ATMs. This process may be interrupted by L-685458.

The aim of the current study was to explore the relationship between IMAAGs and the BRCA tumor microenvironment. The findings showed that the BCPRS and BCRRS scoring systems can be used to comprehensively evaluate the prognosis of OS and PFS in breast cancer patients. Their predictive powers were confirmed using clinical samples. The BCPRS scoring system was independent of the traditional TNM staging, implying that it can be used as a supplementary scoring system for the prognosis of breast cancer. In addition, the findings of this study provide information on the oncogenic and tumor-suppressive functions of MALAT1 in breast cancer cells. In summary, BCPRS and BCPRS-related genes (HEY1, IFNA13, NKX2-3, NR2F1, POU5F1, and YY1) can be used to evaluate the immune microenvironment and tumor purity in breast cancer patients.

Furthermore, neural network-based deep learning models were established to predict breast cancer cell types using BCPRS-related genes (HEY1, IFNA13, NKX2-3, NR2F1, POU5F1, and YY1). A BCPRS-related gene-based neural network showed high accuracy using the training set and the testing set. Therefore, these findings show the importance of BCPRS-related genes in exploring the tumor microenvironment.

Although genetic changes may affect the level of mRNA expression, the findings of this study showed no significant variation in tumor copy number and nucleotide mutations of the six IMAAG genes (HEY1, IFNA13, NKX2-3, NR2F1, POU5F1, and YY1). BCRRS was surprisingly found to be associated with the risk of stroke. These findings show that changes in expression levels of the six IMAAG genes were more likely to arise due to alterations in the tumor microenvironment rather than variations in CNV or SNPs. scRNA-seq and bulk RNA-seq data analysis showed that TNBC cells follow a two-dimensional differentiation trajectory and that their differentiation states are correlated with BCPRS. Adipocytes and adipose tissue macrophages (ATMs) were highly enriched in the high BCPRS cluster. Moreover, drug-ceRNA and ligand-receptor interaction analysis predicted that the LINC00276&MALAT1/miR-206/FZD4-Wnt7b pathway based on BCPRS may help in exploring the mechanism of tumors that lead to mortalities and can provide insights on the development of new drug combinations. BCPRS-related gene-based neural network-based deep learning models showed that these genes have great potential in mapping the tumor microenvironment. These findings provide novel ideas for the identification of high-risk breast cancer and the development of individualized treatment options against the disease in the future.

The BCPRS and BCRRS scoring systems used in the current study showed a potential relationship between the 6 IMAAG genes and the microenvironment of breast cancer. However, further functional experiments should be performed to explore the potential mechanism of action of IMAAG genes. This model should be verified further using independent cohorts to ensure that it is highly robust. In addition, future experiments are needed to explore the underlying mechanisms of the drug-ceRNA network and the potential LINC00276&MALAT1/miR-206/FZD4-Wnt7b pathway.

## 5. Conclusion

In this study, BCPRS and BCRRS scoring systems were established based on six IMAAGs with satisfactory clinical utility. The finding showed that adipocytes and ATMs were highly enriched in the high BCPRS cluster and were associated with poor prognosis. Moreover, ligand-receptor interactions and potential regulatory mechanisms showed that LINC00276&MALAT1/miR-206/FZD4-Wnt7b is a potential pathway in the functions of IMAAGs in breast cancer metastasis and recurrence. In summary, comprehensive evaluation of individual IMAAGs, BCPRS, and BCRRS provides a better understanding of the tumor microenvironment in breast cancer and insights on development of personalized treatment options.

## Figures and Tables

**Table 1:** Demographics and clinicopathological characteristics of BRCA patients in the TCGA cohort in the high and low BCPRS groups.

Variable	BRCA TCGA cohort		
	High BCPRS (*n* = 467)	Low BCPRS (*n* = 467)	*p* value
Survival state			*p* ≤ 0.001^∗∗^
Alive	434(92.93)	457(97.86)	
Dead	33(7.07)	10(2.14)	
Follow-up time (day)	1081.78 ± 967.10	1317.78 ± 1371.82	0.002^∗∗^
Age (year)	58.43 ± 13.10	57.39 ± 12.69	0.216
T			0.034^∗^
T1	118(25.27)	125(26.77)	
T2	266(56.96)	284(60.81)	
T3	71(15.20)	42(8.99)	
T4	12(2.57)	14(3.00)	
NA	0(0.00)	2(0.43)	
N			0.007^∗∗^
N0	220(47.11)	232(49.68)	
N1	150(32.12)	154(32.98)	
N2	45(9.64)	58(12.42)	
N3	43(9.21)	21(4.50)	
NA	9(1.93)	2(0.43)	
M			0.007^∗∗^
M0	371(79.44)	406(86.94)	
M1	6(1.28)	6(1.28)	
NA	90(19.27)	55(11.78)	
Stage			0.699
Stage I	76(16.27)	83(17.77)	
Stage II	268(57.39)	276(59.10)	
Stage III	111(23.77)	96(20.56)	
Stage IV	6(1.28)	4(0.86)	
Stage X	6(1.28)	8(1.71)	
Race			0.151
Asian	33(7.07)	23(4.93)	
Black or African American	86(18.42)	67(14.35)	
White	305(65.31)	331(70.88)	
NA	43(9.21)	46(9.85)	
Treatment			0.556
Pharmaceutical	225(48.18)	234(50.11)	
Radiation	242(51.82)	233(49.89)	
Immunity groups			0.001^∗∗^
High immunity group	131(28.05)	110(23.55)	
Medium immunity group	285(61.03)	267(57.17)	
Low immunity group	51(10.92)	90(19.27)	
StromalScore	710.16 ± 621.54	271.26 ± 651.71	*p* ≤ 0.001^∗∗^
ImmuneScore	887.00 ± 819.86	647.32 ± 861.61	*p* ≤ 0.001^∗∗^
ESTIMATEScore	1597.16 ± 1287.73	918.58 ± 1307.58	*p* ≤ 0.001^∗∗^
TumorPurity	0.66 ± 0.14	0.73 ± 0.13	*p* ≤ 0.001^∗∗^
mRNAsi	0.31 ± 0.09	0.38 ± 0.09	*p* ≤ 0.001^∗∗^
EREG.mRNAsi	0.66 ± 0.10	0.67 ± 0.11	0.126
Relative expression of BCRRS-related genes			
HEY1	16.33 ± 0.89	15.63 ± 1.00	*p* ≤ 0.001^∗∗^
IFNA13	0.59 ± 2.22	0.02 ± 0.37	*p* ≤ 0.001^∗∗^
NKX2.3	5.42 ± 5.10	2.70 ± 4.56	*p* ≤ 0.001^∗∗^
NR2F1	16.59 ± 1.53	15.22 ± 1.42	*p* ≤ 0.001^∗∗^
POU5F1	11.46 ± 1.30	12.42 ± 1.61	*p* ≤ 0.001^∗∗^
YY1	18.18 ± 0.27	18.47 ± 0.31	*p* ≤ 0.001^∗∗^

^∗^*p* < 0.05; ^∗∗^*p* < 0.01.

**Table 2:** Demographics and clinicopathological characteristics of BRCA patients in the clinical cohort in the high and low BCRRS groups.

Variable	BRCA clinical cohort		
	High BCRRS (*n* = 467)	Low BCRRS (*n* = 467)	*p* value
Tumor recurrence			*p* ≤ 0.001^∗∗^
No	57(58.76)	87(89.69)	
Yes	40(41.24)	10(10.31)	
Follow-up time (day)	1804.67 ± 755.72	2314.40 ± 514.56	*p* ≤ 0.001^∗∗^
Age (year)	52.97 ± 13.54	51.76 ± 10.77	0.493
T			0.415
T1	33(34.02)	42(43.30)	
T2	57(58.76)	49(50.52)	
T3	7(7.22)	6(6.19)	
N			0.015^∗^
N0	46(47.42)	60(61.86)	
N1	21(21.65)	23(23.71)	
N2	23(23.71)	7(7.22)	
N3	7(7.22)	7(7.22)	
Grade			0.032^∗^
G1	1(1.03)	6(6.19)	
G2	51(52.58)	60(61.86)	
G3	45(46.39)	31(31.96)	
Pausimenia			0.885
No	44(45.36)	45(46.39)	
Yes	53(54.64)	52(53.61)	
Relative expression of BCRRS-related genes (normalized with *z*-score)			
HEY1	0.34 ± 0.88	−0.34 ± 1.00	*p* ≤ 0.001^∗∗^
IFNA13	0.22 ± 1.38	−0.22 ± 0.02	0.003^∗∗^
NKX2-3	0.22 ± 0.99	−0.22 ± 0.97	0.002^∗∗^
NR2F1	0.43 ± 0.95	−0.43 ± 0.85	*p* ≤ 0.001^∗∗^
POU5F1	−0.38 ± 0.68	0.38 ± 1.12	*p* ≤ 0.001^∗∗^
YY1	−0.43 ± 0.86	0.43 ± 0.94	*p* ≤ 0.001^∗∗^

^∗^*p* < 0.05; ^∗∗^*p* < 0.01.

**Table 3:** Comprehensive multifactorial COX analysis of BRCA demographics, pathological characteristics, and microenvironment.

Variables	Overall survival (OS)	
	HR(95% CI)	*p* value
BCPRS	7.34(3.627~14.853)	*p* ≤ 0.001
Age	1.046(1.018~1.075)	0.001
mRNAsi	137.5(0.411 ~ 4.60 × 10^4^)	0.097
	3.484(0.120~101.498)	0.468
T	1.377(0.950~1.997)	0.092
N	1.414(1.045~1.914)	0.025
M	1.101(0.726~1.669)	0.651
Race	1.073(0.746~1.542)	0.705
StromalScore	1.002(0.999~1.004)	0.162
TumorPurity	97.67(0.000 ~ 4.56 × 10^12^)	0.715
ImmuneScore	1(0.997~1.003)	0.982

**Table 4:** C-index of breast OS and PFS prediction models.

Dataset group	C-index of the OS prediction model		C-index of the PFS prediction model	
	C-index	The C-index (95% CI)	C-index	The C-index (95% CI)
Training cohort	0.802	0.709-0.895	0.864	0.784-0.944
Validation cohort	0.747	0.600-0.894	0.793	0.672-0.914
Entire cohort	0.767	0.681-0.853	0.843	0.776-0.909

## Data Availability

The datasets generated and analyzed during the present study are available from the corresponding author on reasonable request.

## Abbreviations

ARGS: Autophagy-related genes
ATMs: Adipose tissue macrophages
BCPRS: Breast Cancer Prognostic Risk Score
BCRRS: Breast Cancer Recurrence Risk Score
BPs: Biological processes
CCs: Cellular components
CNV: Copy Number Variation
CS: Conditional Survival
DCA: Decision curve analysis
DEGs: Differentially Expressed Genes
DEMs: Differentially expressed mRNAs
GO: Gene Ontology
GSEA: Gene Set Enrichment Analysis
GSVA: Gene Set Variation Analysis for Microarray and RNA-seq data
HNC: Head and neck cancer
IMAAGs: Immune, Methylated, and Autophagy-Associated Genes
KEGG: Kyoto Encyclopedia of Genes and Genomes
K-M: Kruskal-Wallis
LASSO: Least absolute shrinkage and selection operator
MFs: Molecular functions
miRNA: Micro-RNA
OS: Overall survival
PCA: Principal component analysis
PFS: Progression-free survival
PPI: Protein-protein interaction
qPCR: Quantitative real-time PCR
ROC: Receiver operating characteristic
SNP: Single Nucleotide Polymorphism
TCGA: The Cancer Genome Atlas
TNBC: Triple-negative breast cancer
TOM: Topological Overlap Measure
WGCNA: Weighted Gene Coexpression Network Analysis
UMAP: Uniform Manifold Approximation and Projection.

## Additional Points

*Study Summary*. Although a highly coordinated interaction between Immune, Methylated, and Autophagy-Associated Genes (IMAAGs) exists, their comprehensive application as specific markers for analysis of the tumor microenvironment and prediction of the prognosis of breast cancer has not been explored. Six prognostic IMAAGs were identified to comprehensively explore prognosis of OS and PFS in breast cancer. The findings showed that the BCPRS and BCRRS scoring systems based on 6 IMAAGs can accurately stratify the prognosis of breast cancer patients. OS and PFS nomogram prediction models were constructed with high clinical value. Analysis showed that BCRRS was associated with the risk of stroke. Protein-protein interaction (PPI) and drug-ceRNA networks based on the differences in the Breast Cancer Prognostic Risk Score (BCPRS) were constructed. Moreover, adipocytes and adipose tissue macrophages (ATMs) were highly enriched in the high BCPRS cluster and were associated with poor prognosis. Ligand-receptor interactions and potential regulatory mechanisms were explored and the LINC00276&MALAT1/miR-206/FZD4-Wnt7b pathway was identified to play an important role in the functions of these genes and can be used to explore targets against breast cancer metastasis and recurrence. Furthermore, neural network-based deep learning models were established to predict cell composition using BCPRS gene signatures.
