# Supplementary material for: Roles of the Immune/Methylation/Autophagy Landscape on Single-Cell Genotypes and Stroke Risk in Breast Cancer Microenvironment
Source: Oxid Med Cell Longev. 2021 Aug 19;2021:5633514. doi: 10.1155/2021/5633514 (PMC8397558; doi:10.1155/2021/5633514)
Supplement: Supplementary Materials — Supplementary tables and figures. [file 5633514.f1.pdf]

SUPPLEMENTARY FIGURES

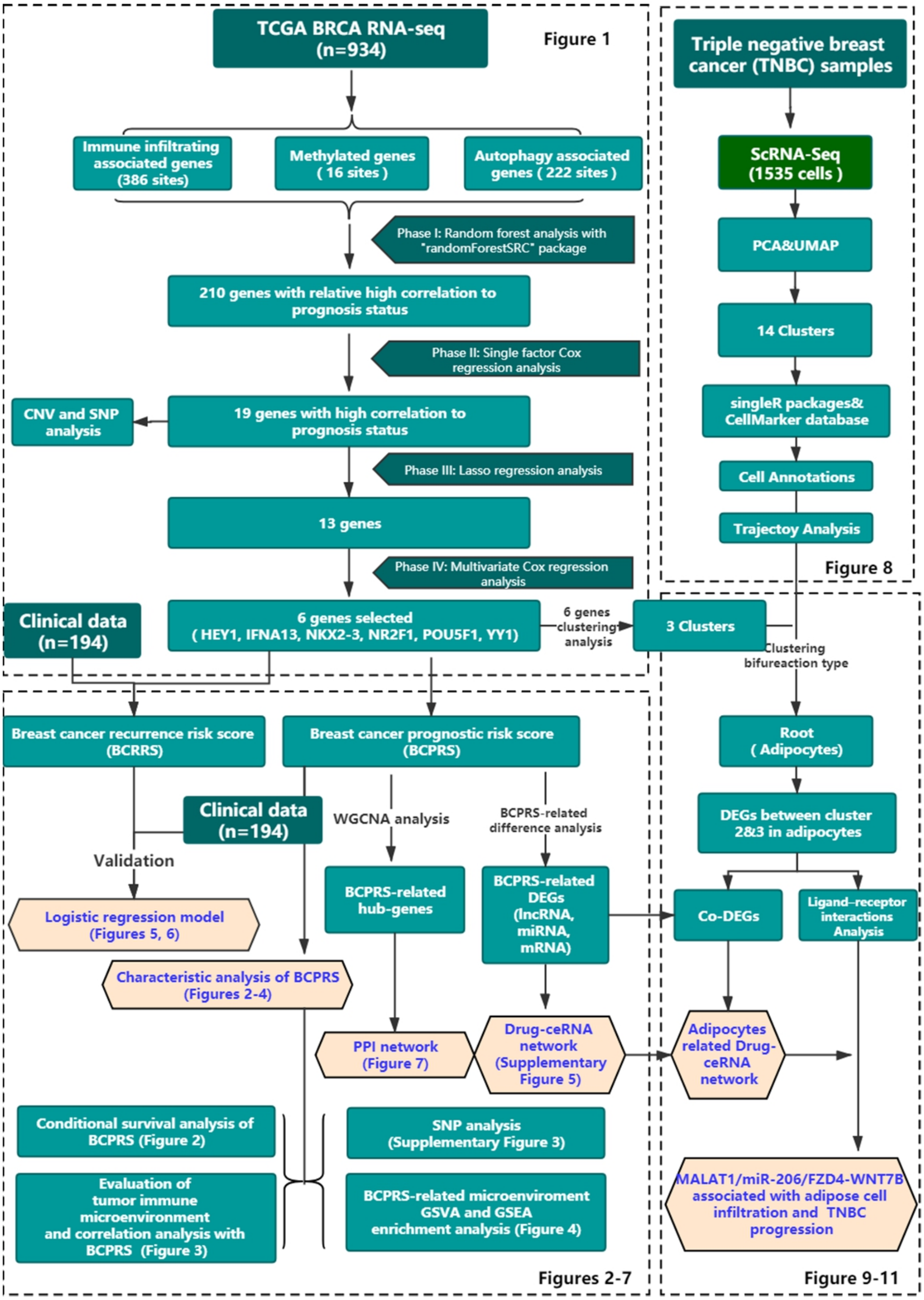

Supplementary Figure 1. Schematic diagram showing the study design and principal findings.

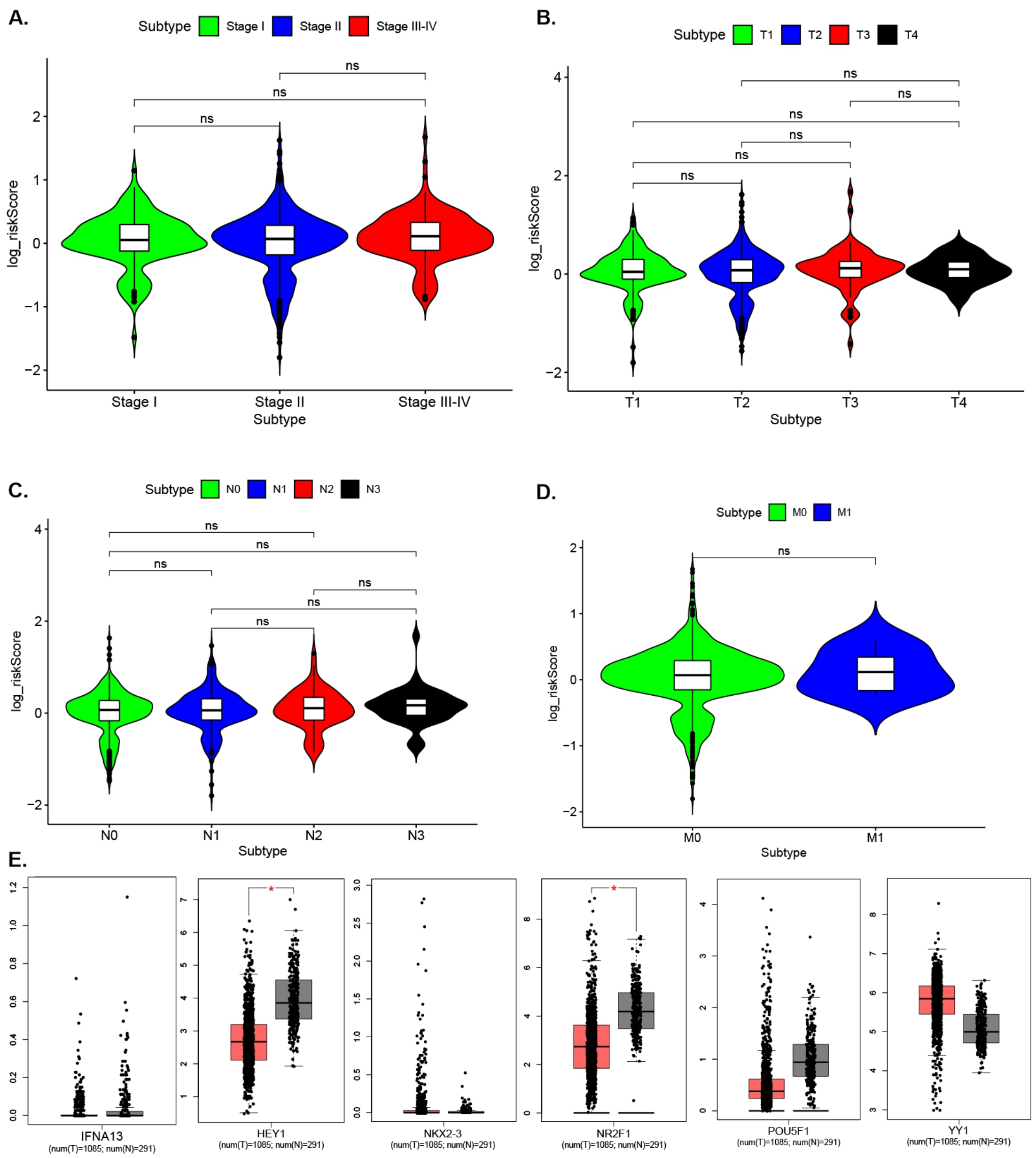

Supplementary Figure 2.

A-D. Violin figures suggested that there were no significant differences between the BCPRS and TNM stages (P>0.05);

E. Relative expression of IFNA13, HEY1, NKX2-3, NR2F1, POU5F1 and YY1 in samples from TCGA and GTEX cohorts.

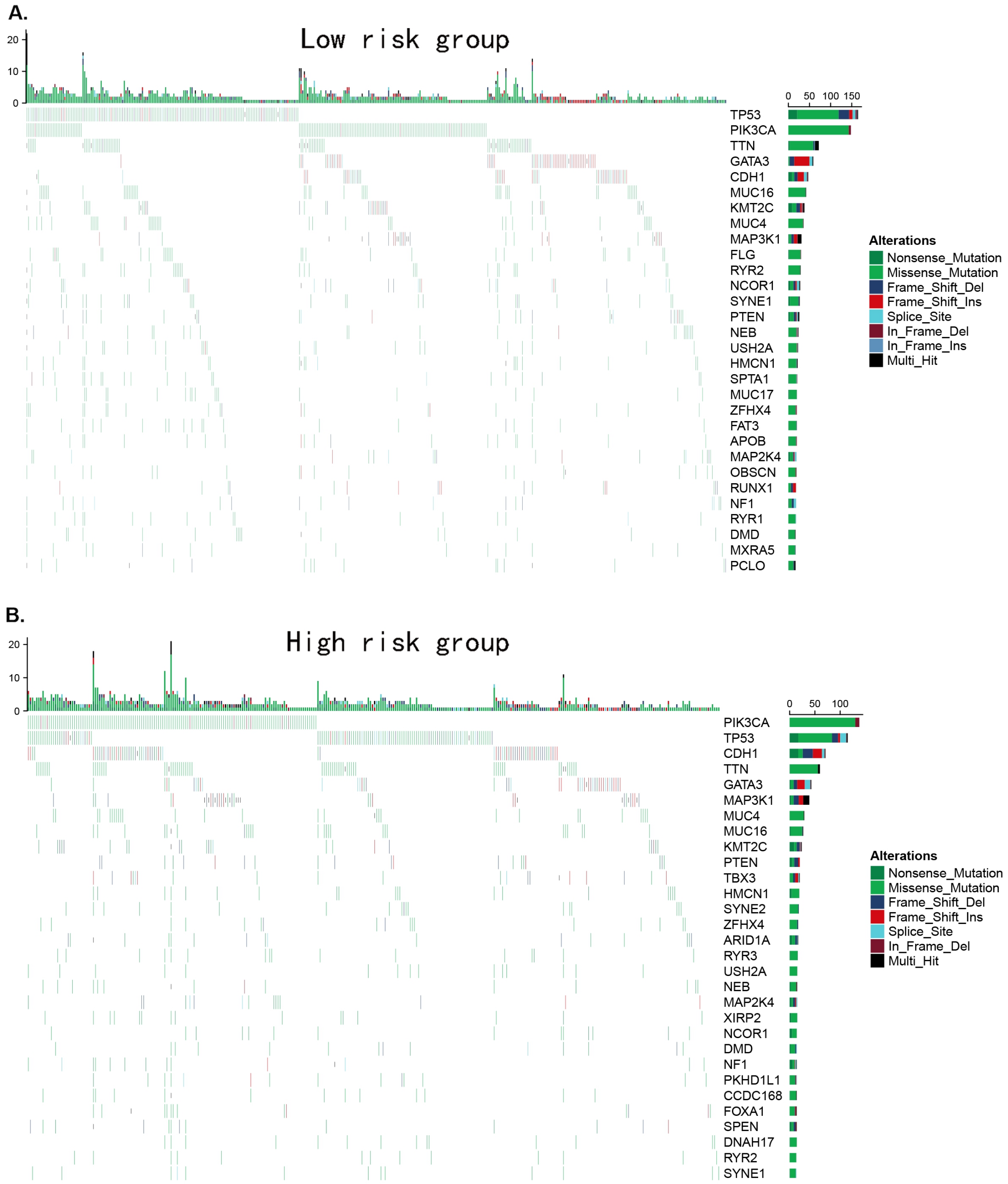

Supplementary Figure 3.

The waterfall plot of tumor somatic mutations in patients with (A) low BCPRS and (B) high BCPRS. The figure on the right indicates the frequency of mutations in each gene.

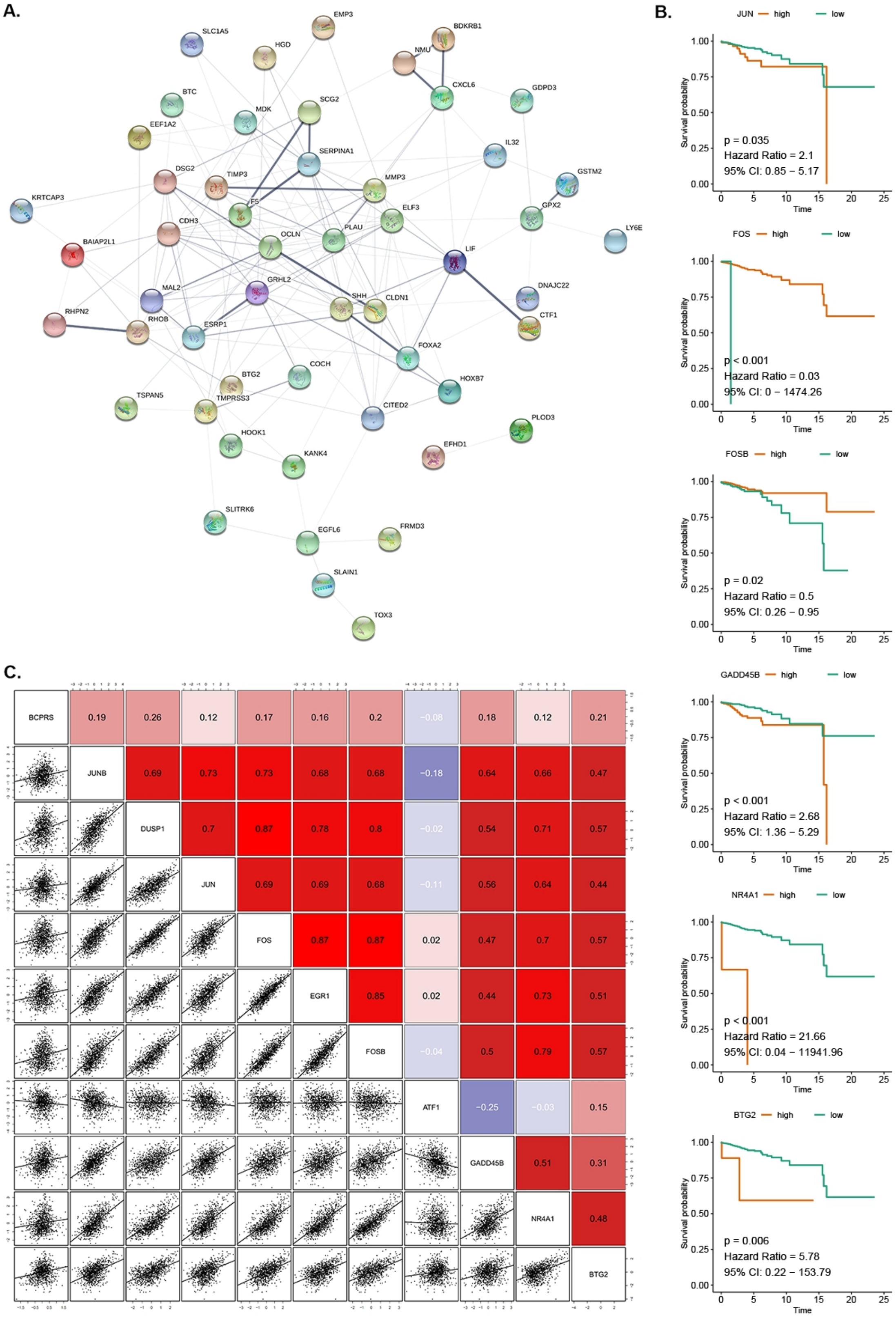

Supplementary Figure 4.

A. Construction of a Protein-protein Interaction (PPI) network of genes from the black module with a PPI enrichment P value of 1.0e-16;

B. K-M curves of overall survival in hub genes (JUN, FOS, FOSB, GADD45B, NR4A1 and BTG2) with a significant difference (P<0.05);

C. Correlations among hub genes (FOSB, JUNB, EGR1, GADD45B, JUN, NR4A1, BTG2, ATF3, FOS and DUSP1) in the black module (TCGA-BRCA cohort, n= 934)

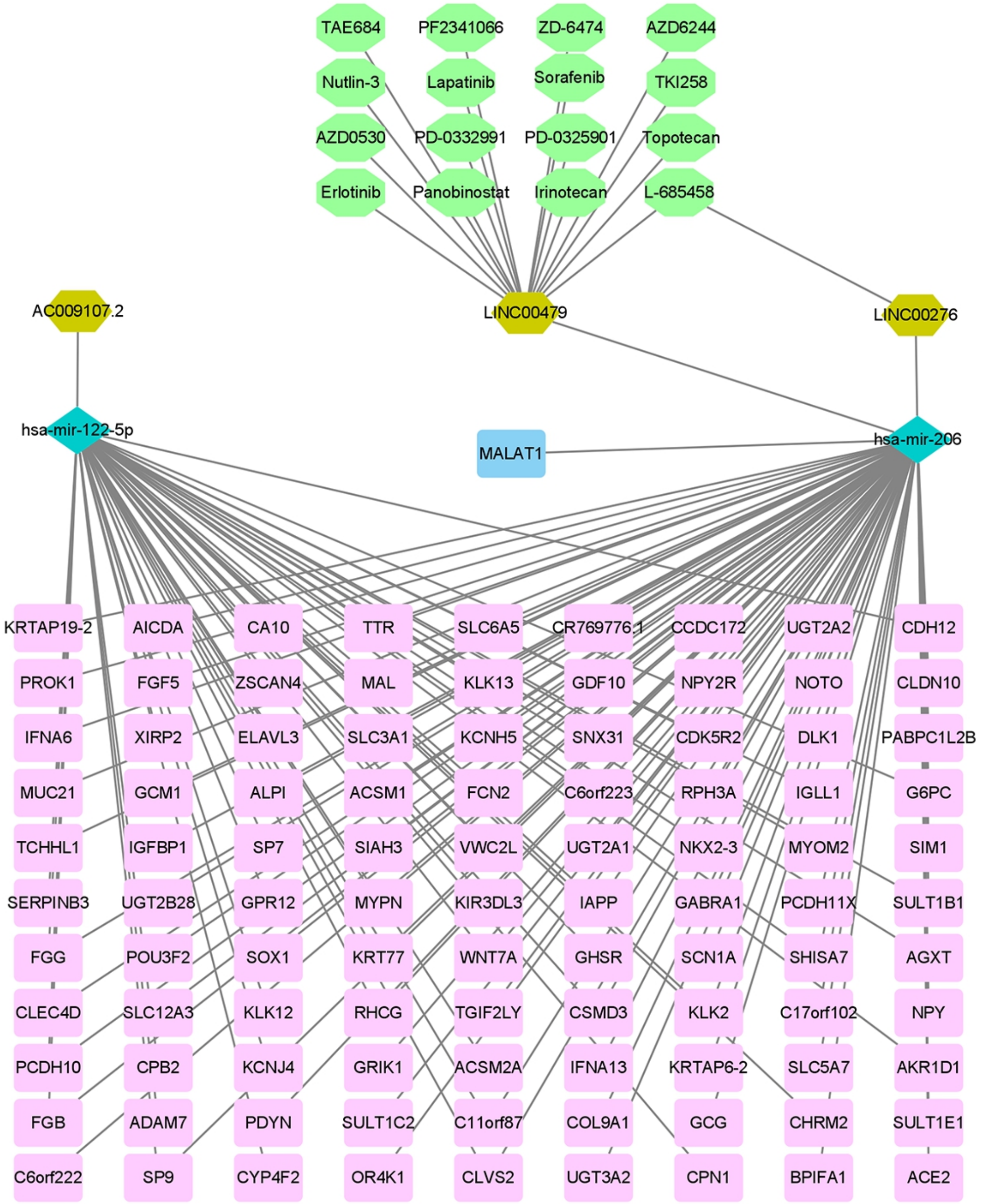

Supplementary Figure 5.

A-B. Cell ontology class plot of mammary gland from Tabula Muris;

C-D. The expression of six BCPRS-related genes (YY1, POU5F1, NKX2-3, NR2F1,

HEY1 and IFNA13) in scRNA-seq is shown with droplet method (C) and FACS method (D). Blue represents high expression and gray represents low expression.

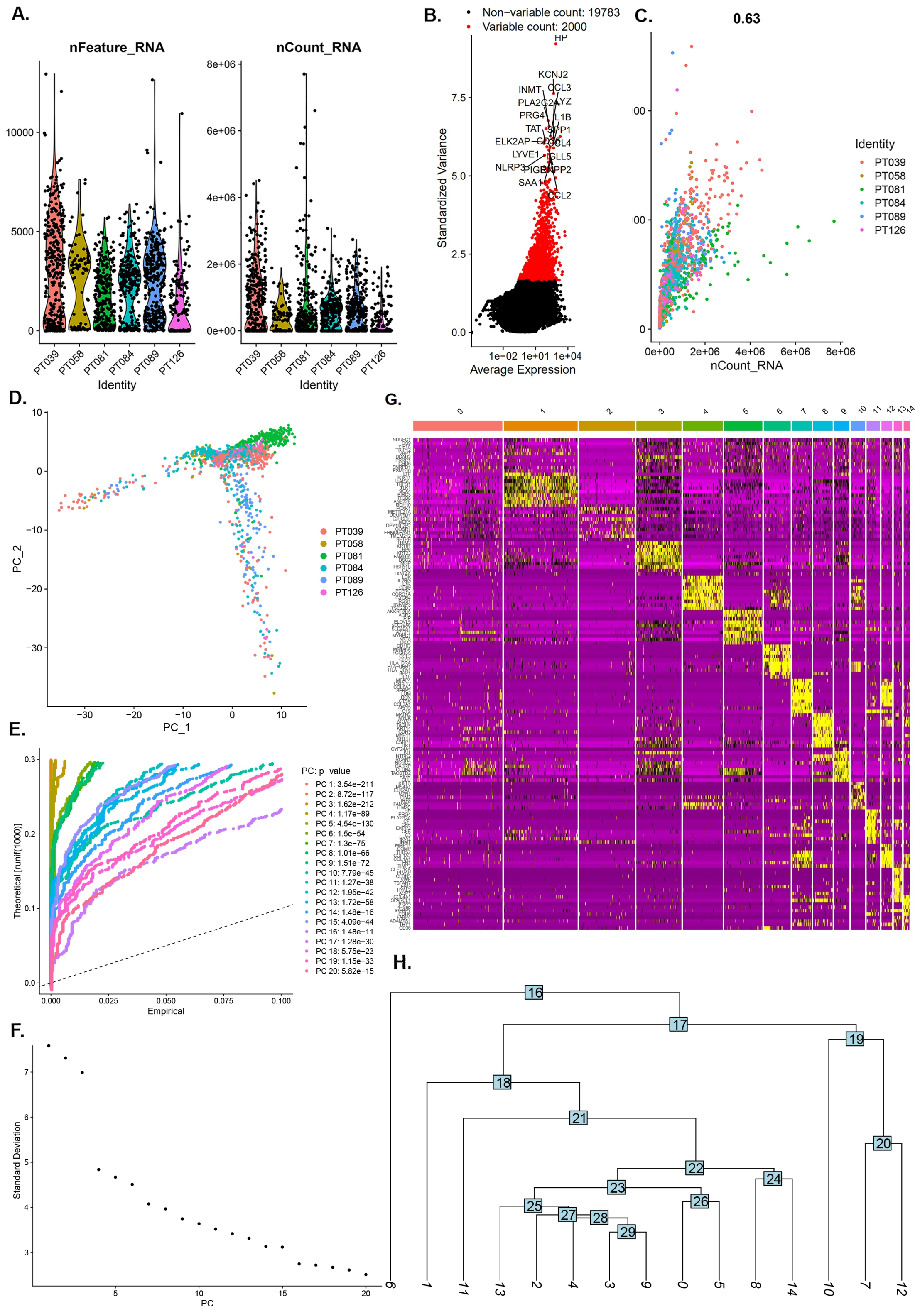

Supplementary Figure 6. RNA-seq data based on breast cancer TNBC single cells

reveal the high heterogeneity of breast cancer TNBC tumor cells.

A. After quality control of 1,535 cells in the tumor core of six human TNBC specimens, 1,266 cells were included for analysis;

B. ANOVA plots revealed 1,783 corresponding genes in all cells of TNBC. Red dots indicate highly variable genes and black dots represent non-variable genes. The top 20 marker genes for each cell cluster are labeled in the figure;

C. The number of detected genes is significantly correlated with sequencing depth, with a Pearson correlation coefficient of 0.63;

D. PCA did not show clear separation of these TNBC cells;

E-F. PCA identified 20 PCs with an estimated P value < 0.05;

G-H. The top 20 marker genes for each cell cluster are revealed in the heat map (G). The clustering of different cell clusters is shown in Figure (H).

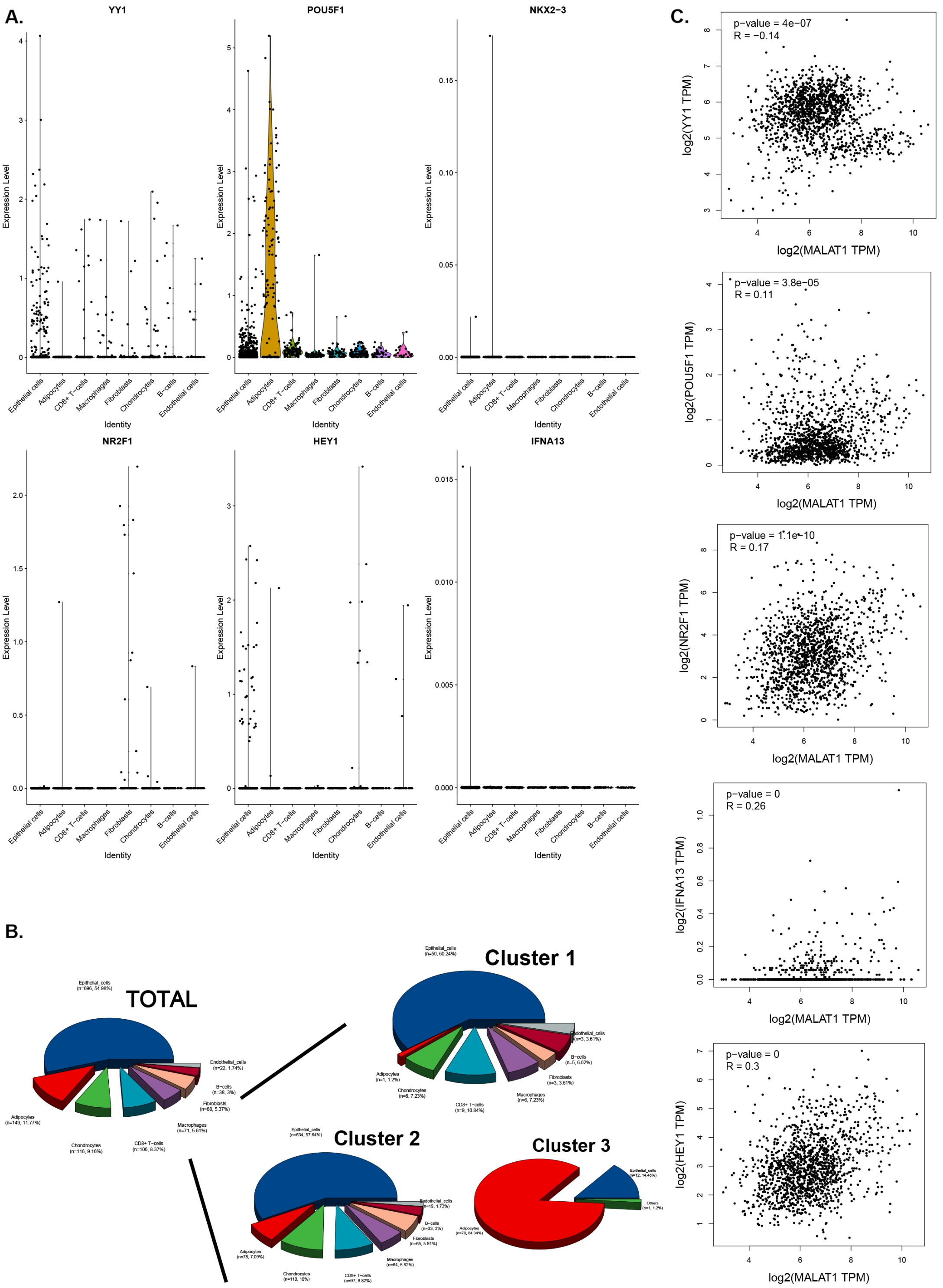

Supplementary Figure 7.

A. The expression of six BCPRS-related genes (YY1, POU5F1, NKX2-3, NR2F1, HEY1 and IFNA13) in different types of cells is shown;

B. Three cellular clusters were obtained based on 6 BCPRS-related gene clustering.

Composition of the proportions of cells in the different clusters;

C. The correlation analysis demonstrated that the expression of MALAT1 and other genes (YY1, POU5F1, NR2F1, IFNA13 and HEY1) was significantly correlated in TCGA BRCA data.

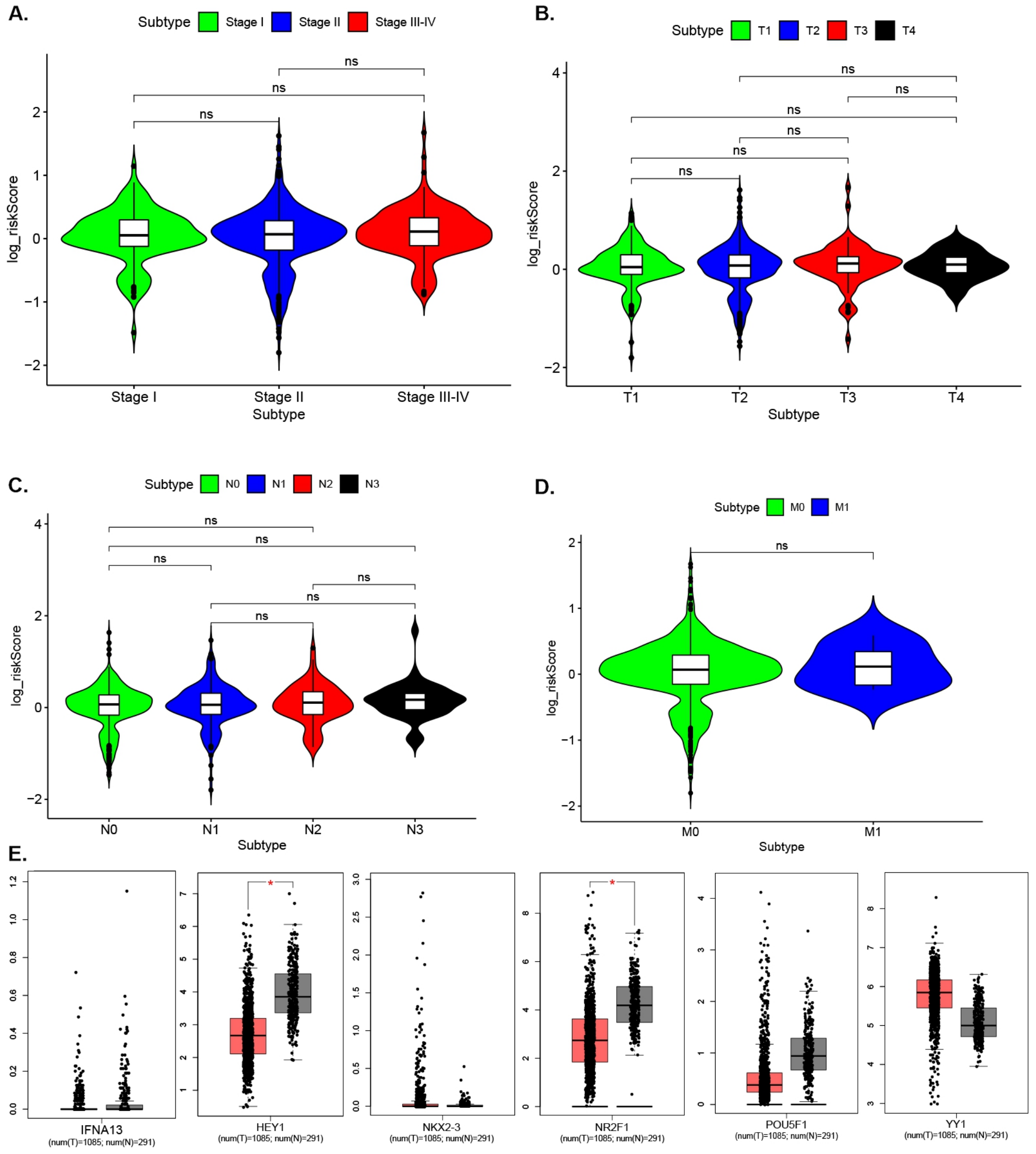

Supplementary Figure 8. Construction of the Drug-ceRNA network based on BCPRS.

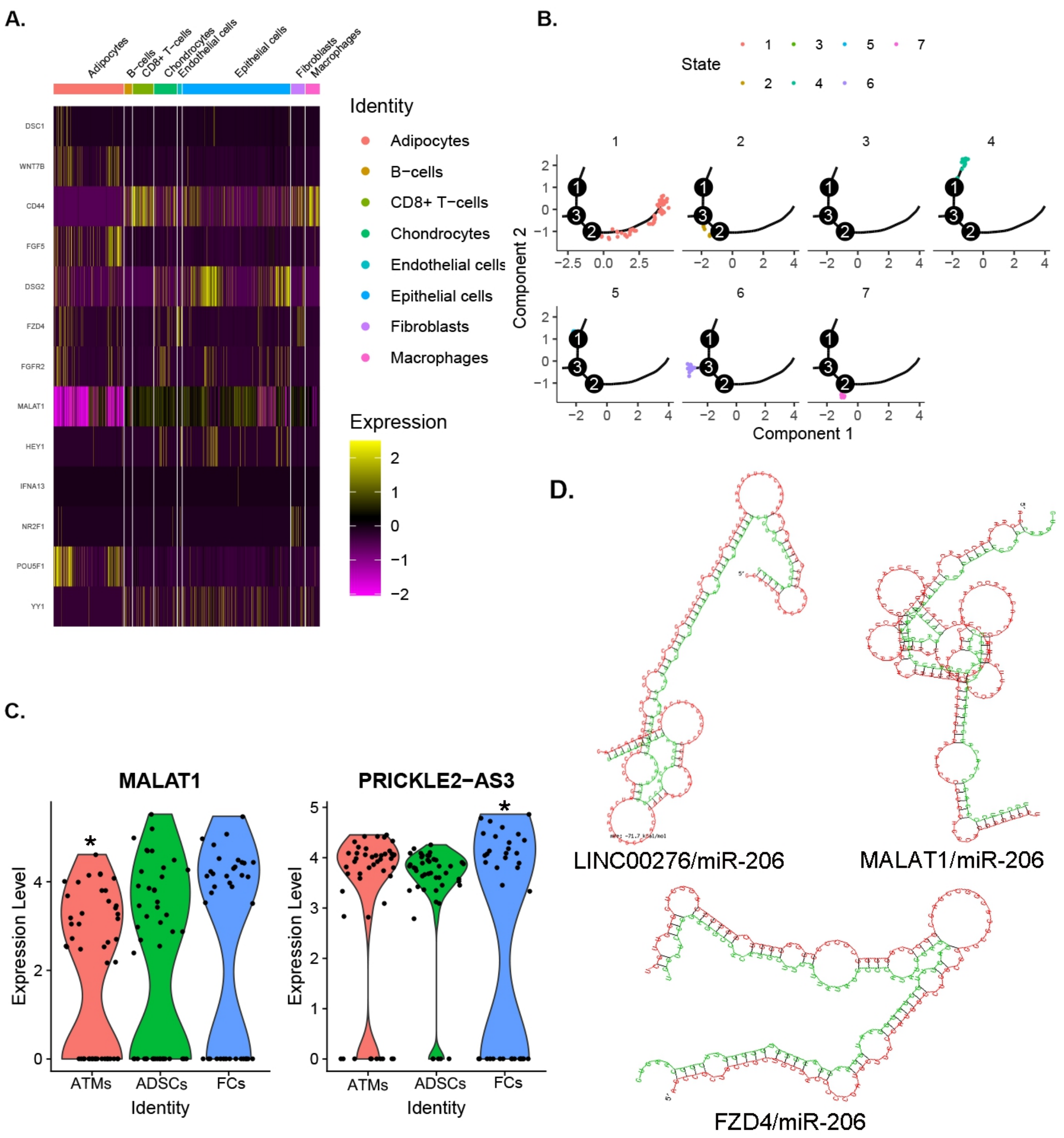

Supplementary Figure 9.

A. The expression of the screened genes (DSC1, Wnt7b, CD44, FGF5, DSG2, FZD4, FGFR2, MALAT1, HEY1, IFNA13, NR2F1, POU5F1 and YY1) in different cell types is shown in a heat map;

B. A faceted plot of the distribution of the cell's proposed temporal trajectory. State indicates sequentially increasing proposed temporal sequence;

C. Expression level of MALAT1 and PRICKLE2-AS3 in adipose tissue macrophages (ATMs), adipose-derived stem cells (ADSCs), fat cells (FCs) (*, $p<0.05$);

D. Bioinformatics prediction of miR-206 as the target miRNA of LINC00276

(mfe:-62.5 kcal/mol) and MALAT1 (mfe:-71.7kcal/mol) as well as FZD4 (mfe:-72.3kcal/mol) using RNAhybrid 2.12. MFE: Minimum free energy.

Supplementary Table 1. Desposited Data

Breast cancer data from the author's hospital (n=194)	Shanghai General Hospital
TCGA-BRCA RNA-seq files	Genomic Data Commons
TCGA-BRCA Copy Number Variation data	Genomic Data Commons
TCGA-BRCA Single Nucleotide Polymorphism data	Genomic Data Commons
TCGA gene expression data	Genomic Data Commons
TCGA patients clinic data	Genomic Data Commons
Single cell sequencing data of TNBC	GSE118389 from GEO database
Curated receptors, ligands and their interactions data	CellPhoneDB repository
The correlation data between lncRNA expression levels and the IC50 values of drugs.	LncMAP Project Consortium
The interaction data between DE-lncRNAs and DE-miRNAs	The miRcode database
The interaction data between DE-mRNAs and DE-miRNAs	The miRWalk3.0 database and the miRTarBase (Version 7.0)

Supplementary table 2. Multivariable Cox model of OS for BRCA

Variables	Overall survival (OS)	
	HR(95%CI)	*p-value*
Age	1.035[1.009 ~ 1.062]	0.008
T	1.377[0.956 ~ 1.983]	0.085
N	1.385[1.031 ~ 1.862]	0.031
M	1.01[0.673 ~ 1.517]	0.960
BCPRS	8.488[4.112 ~ 17.522]	0.000

Supplementary table 3. Multivariable Cox model of PFS for BRCA

Variables	Overall survival (OS)	
	HR(95%CI)	*p-value*
Age	1.011[0.989 ~ 1.034]	0.337
T	0.897[0.544 ~ 1.478]	0.669
N	2.162[1.624 ~ 2.877]	0.000
Grade	1.09[0.627 ~ 1.896]	0.759
BCRRS	9.687[4.909 ~ 19.115]	0.000
